# Genomic Insights into Hybridization and Speciation of Mitten Crabs in the *Eriocheir* Genus

**DOI:** 10.1093/gpbjnl/qzaf079

**Published:** 2025-09-15

**Authors:** Jun Wang, Xin Hou, Xiaowen Chen, Roland Nathan Mandal, Nusrat Hasan Kanika, Chunhong Yuan, Yongju Luo, Chenghui Wang

**Affiliations:** Key Laboratory of Freshwater Aquatic Genetic Resources Certificated by the Ministry of Agriculture and Rural Affairs / National Demonstration Center for Experimental Fisheries Science Education / Shanghai Engineering Research Center of Aquaculture, Shanghai Ocean University, Shanghai 201306, China; Key Laboratory of Freshwater Aquatic Genetic Resources Certificated by the Ministry of Agriculture and Rural Affairs / National Demonstration Center for Experimental Fisheries Science Education / Shanghai Engineering Research Center of Aquaculture, Shanghai Ocean University, Shanghai 201306, China; Key Laboratory of Freshwater Aquatic Genetic Resources Certificated by the Ministry of Agriculture and Rural Affairs / National Demonstration Center for Experimental Fisheries Science Education / Shanghai Engineering Research Center of Aquaculture, Shanghai Ocean University, Shanghai 201306, China; Key Laboratory of Freshwater Aquatic Genetic Resources Certificated by the Ministry of Agriculture and Rural Affairs / National Demonstration Center for Experimental Fisheries Science Education / Shanghai Engineering Research Center of Aquaculture, Shanghai Ocean University, Shanghai 201306, China; Key Laboratory of Freshwater Aquatic Genetic Resources Certificated by the Ministry of Agriculture and Rural Affairs / National Demonstration Center for Experimental Fisheries Science Education / Shanghai Engineering Research Center of Aquaculture, Shanghai Ocean University, Shanghai 201306, China; Faculty of Agriculture, Agri-Innovation Center, Iwate University, Morioka 020-8550, Japan; Guangxi Tilapia Genetic Breeding Center, Guangxi Academy of Fishery Sciences, Nanning 530021, China; Key Laboratory of Freshwater Aquatic Genetic Resources Certificated by the Ministry of Agriculture and Rural Affairs / National Demonstration Center for Experimental Fisheries Science Education / Shanghai Engineering Research Center of Aquaculture, Shanghai Ocean University, Shanghai 201306, China

**Keywords:** Hybridization, Speciation, Reproductive isolation, Ecological distribution, Adaptative radiation

## Abstract

Hybridization is a prominent and influential phenomenon with significant implications for adaptive evolution, species distribution, and biodiversity. However, the intricacies of how hybridization influences genomic structure and facilitates adaptive evolution remain poorly understood. By analyzing whole-genome data from seven populations within the *Eriocheir* genus across diverse geographic regions, we validated a complex hybridization history between Chinese and Japanese mitten crabs. This hybridization gave rise to two distinct ecological species: Hepu and Russian mitten crabs with unique genomic architectures and adaptations. Genes related to reproduction, development, and temperature adaptation exhibited divergent selection signals, potentially contributing to their phenotypic diversity and ecological niches. Meanwhile, genes associated with reproduction, namely *Birc6*, *Bap31*, and *Poxn*, displayed robust evidence of selective sweeps in Hepu mitten crab. Notably, the favored alleles for these genes originated from the parental lineages during the hybridization process. Furthermore, Hepu mitten crab is a homoploid hybrid species that originated from an ancient hybridization event, resolving its longstanding taxonomic controversy. Our study sheds light on the evolutionary history of mitten crabs and highlights the crucial role of hybridization in driving adaptation, range expansion, and diversification within the *Eriocheir* genus.

## Graphical abstract

**Figure:**
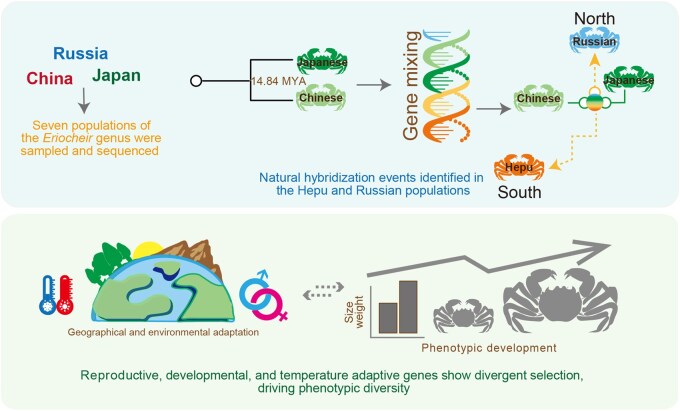

## Introduction

Environmental factors such as climate, water chemistry, and food resources often exert strong selection pressure on environmental adaptation and constrain the geographic distributions of species [1–5]. Hybridization between species, subspecies, or diverged populations serves as a potent driver of increased genetic diversity, reduced genomic vulnerability, and accelerated adaptive evolution, promoting both speciation and radiation [6–12]. Increased genetic diversity through hybridization can promote the natural geographic range expansion and novel genomic architectures in hybrids [13]. However, the full extent of hybridization’s role in global species diversity and the specific contribution of admixed genomes to genomic architecture for adaptive evolution remain the subjects of debate and ongoing research [14–16].

It is evident that hybridization and introgression in hybrid zones promote phenotypic and genotypic adaptations [14,16]. This process is considered a potent force for speciation [17–19]. After hybridization, certain genes may be selected, introducing advantageous foreign alleles that merge into the genetic backgrounds of the parental populations. This process can consequently facilitate geographical expansion and promote adaptive radiation [20–22]. Numerous empirical studies across various animal groups, including butterflies, fishes, birds, and mammals, have substantiated the prevalence of homoploid hybrid speciation [18,19,23–26], suggesting that the increase of species diversity through hybrid speciation may be widespread and hence more imperative for the global species radiation [19,27].

Taxonomy of *Eriocheir* species, comprising *Eriocheir sinensis* (H. Milne Edwards, 1854) (Chinese mitten crab), *Eriocheir japonica* (De Haan, 1835) (Japanese mitten crab), and *Eriocheir hepuensis* (Dai, 1991) (Hepu mitten crab), has long been controversial due to their morphological similarities and limited molecular evidence [28–30]. Especially in the case of *E. hepuensis*, there is ongoing debate regarding whether it should be classified as a subspecies of *E. japonica*, an independent species, or a hybrid [30–36]. In contrast, earlier studies have long treated *E. sinensis* and *E. japonica* as distinct species [31,36]. Mitten crabs are native to East Asia: *E. sinensis* is mainly distributed in the Liao River, Yellow River, and Yangtze River from the northern to the middle part of China [35]. *E. japonica* inhabits the northern East Coast of Korea and Japan [33,37], and *E. hepuensis* is limited to the Nanliu River in southern China [28]. Additionally, invasive mitten crab species, *E. sinensis* and *E. japonica*, have been reported in Europe and North America [30,38–41], while *E. hepuensis* has been established in Iraq and Iran [42,43]. However, due to the difficulty in classifying the *Eriocheir* species only by subjective morphology characters, the exact invasive species of *Eriocheir* in non-native regions remains obscure [30,42]. Recent studies suggest that invasive *Eriocheir* species in Europe may be hybrids [41,44] and that previously identified *E. sinensis* in European waters was molecularly determined to be *E. japonica* [41], underscoring the need to establish the taxonomic status of the *Eriocheir* genus and clarify the origin of *E. hepuensis* through robust genomic data.

*E. sinensis*, an indigenous species in China, is one of the most economically significant freshwater species, supporting a vast aquaculture industry in the country. It is widely cultured in the Yangtze River region, whereas *E. japonica* and *E. hepuensis* have not been cultured as extensively [28]. Previous studies reported that the natural hybridization events occurred between *E. sinensis* and *E. japonica*. Two hybrid zones exist between *E. sinensis* and *E. japonica*: Vladivostok, Russia and the Min River, China [35,45]. Meanwhile, research suggests that *E. hepuensis* in the Nanliu River (Hepu, Guangxi Zhuang Autonomous Region, China) may also be a hybrid [30]. Hybridization events across varying latitudes result in distinct ecological hybrids that inhabit different ecological niches (southern *vs.* northern), although they have the same parental species (*E. sinensis* and *E. japonica*). This fascinating mitten crab system gives us a unique opportunity to investigate the influence of interspecies hybridization on local environmental adaptation and ecological distribution.

In this study, we combined our previously published chromosome-level reference genome of *E. sinensis* [46] with the two *de novo* assembled draft genomes of *E. japonica* and *E. hepuensis* to delve into the intricate world of mitten crab hybridization. Our research aims to investigate the genome characteristics, population structures, genetic variation, and hybridization history of seven mitten crab populations in the *Eriocheir* genus across East Asia. Specifically, we seek to explore how gene flow between representative Chinese, Japanese, Russian, and Hepu mitten crabs have led to new speciation events, driven by genetic mixing. By focusing on physiological and phenotypic differences among these populations, we aim to assess how hybridization contributes to environmental adaptation and speciation. Furthermore, we intend to elucidate the genetic mechanisms behind hybrid genome evolution and their role in adaptation to distinct ecological environments and distribution patterns.

## Results

### Genome assembly and annotation comparison of *Eriocheir* species

High-quality chromosome-level genome of *E. sinensis* was assembled in our previous study [46]. The genomes of *E. japonica* and *E. hepuensis* were sequenced and *de novo* assembled in this study (Tables S1 and S2). The assembled genome sizes were 1.24 Gb for *E. japonica* and 1.18 Gb for *E. hepuensis*, with N50 lengths of 442,749 bp and 122,458 bp, respectively (Table S3). Using prediction evidence and RNA sequencing (RNA-seq) data, 18,418 protein-coding genes were identified in *E. japonica* and 19,253 in *E. hepuensis* which are comparable with those in *E. sinensis* (20,286 protein-coding genes) (Tables S3 and S4).

### Geographical and morphological diversity in mitten crab populations

**Figure 1:**
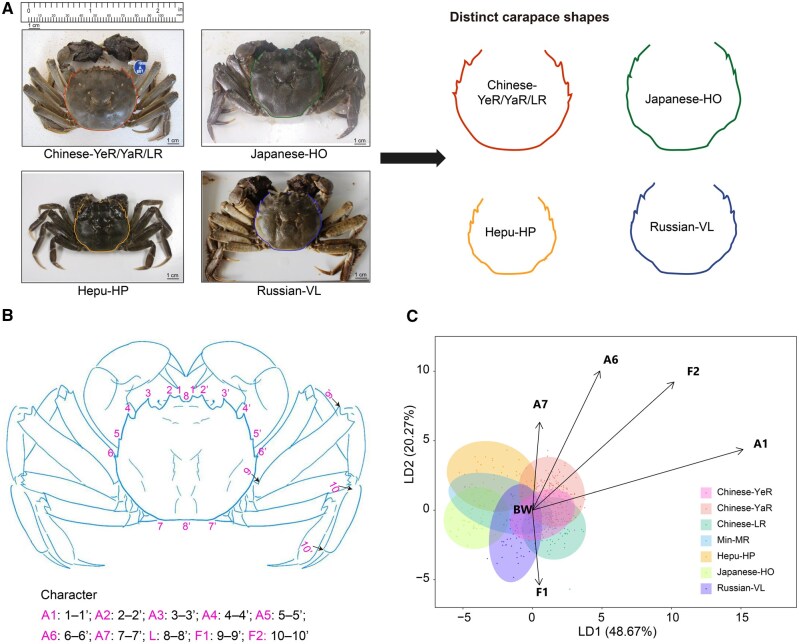
Phenotypic variation among mitten crab populations **A**. Feature images of mitten crabs. **B**. Diagram illustrating the external morphological measurement scheme for the mitten crab. Measured traits include body weight (BW), shell length (L), shell width (A6), length of frontal teeth (A1–A5), and limb length (F1 and F2). Paired numeric labels correspond to the start and end points for each measurement. **C**. LDA based on phenotypic measurements of mitten crabs collected from seven populations. LDA, Linear Discriminant Analysis; LD1/2, the first/second linear discriminant component; YeR, Yellow River; YaR, Yangtze River; LR, Liao River; MR, Min River; HP, Hepu; HO, Hokkaido; VL, Vladivostok.

Mitten crab samples were collected from seven geographic locations across different latitudes, including: Chinese mitten crab collected from the Liao River (LR, China), the Yellow River (YeR, China), and the Yangtze River (YaR, China); mitten crab collected from the Min River (MR, China); Russian mitten crab sourced from Vladivostok (VL, Russia); Japanese mitten crab obtained from Hokkaido (HO, Japan); and Hepu mitten crab collected from Hepu (HP, China) (Table S5). Among these populations, four exhibited distinct morphological differences in body weight and shape (**Figure 1**A and B, Figure S1). As shown in Figure 1A, the Hepu-HP mitten crab population is notably smaller in carapace and body size than the others. Chinese-LR, Chinese-YeR, and Chinese-YaR mitten crabs have nearly circular carapaces with deeper frontal teeth (A1), conspicuous lateral teeth, greater carapace width (A6), and longer limbs (F1 and F2) (Figure 1A and B, Figure S1). In contrast, the Japanese-HO mitten crab population has nearly square carapaces with blunt frontal teeth (A1), less prominent lateral teeth, narrower carapace width (A6), and shorter limbs (F1 and F2) (Figure 1A and B, Figure S1).

The Analysis of Variance (ANOVA) results further showed that southern populations, Hepu-HP and Min-MR mitten crabs have the smallest adult body weight **(**Figure S1**)**. In the Japanese-HO mitten crab, width of first pair of frontal teeth (A1), carapace width (A6), femur length (F1), and tibia length (F2) were the smallest, while these traits were the largest in the Chinese mitten crab (Figure S1). As for Hepu-HP and Russian-VL mitten crabs, we identified A1, A6, F1, and F2 to show intermediate levels compared with Chinese-LR, Chinese-YeR, Chinese-YaR, and Japanese-HO mitten crabs, consistent with previous research (Figure S1) [45]. However, Russian-VL mitten crab presents a larger body weight, and smaller A1 and A6 compared with Hepu-HP mitten crab (Figure S1). Additionally, Linear Discriminant Analysis (LDA) revealed distinct phenotypic clusters among the seven populations, with the first linear discriminant component (LD1) separating Chinese-LR, Chinese-YeR, and Chinese-YaR from Japanese-HO mitten crabs, indicating distinct phenotype variation (Figure 1C).

Moreover, the time required for sexual maturation varies among the mitten crab populations from different regions (Figure S2A). Hepu mitten crab, located in a high-temperature zone, reaches sexual maturity within one year. In contrast, Japanese and Chinese mitten crabs require two years, while Russian mitten crab, found in a lower-temperature zone, takes three years to mature, reflecting the influence of regional temperature on their maturation periods (Figure S2B).

### Ancestral history of populations within the *Eriocheir* genus

We conducted genome resequencing and variant discovery based on 139 samples of mitten crabs at the whole-genome level using *E. sinensis* chromosomal-level genome as reference [46]. We obtained a total of 5547.87 Gb of raw data, with an average of 39 Gb (∼ 23× depth) per individual (Table S6). After quality filtering, all the sequenced reads were mapped to the *E. sinensis* reference genome, the average mapping rate was above 97% (Table S6). We identified a total of 10.27 million high-quality single-nucleotide polymorphisms (SNPs) after filtration (Table S7).

**Figure 2:**
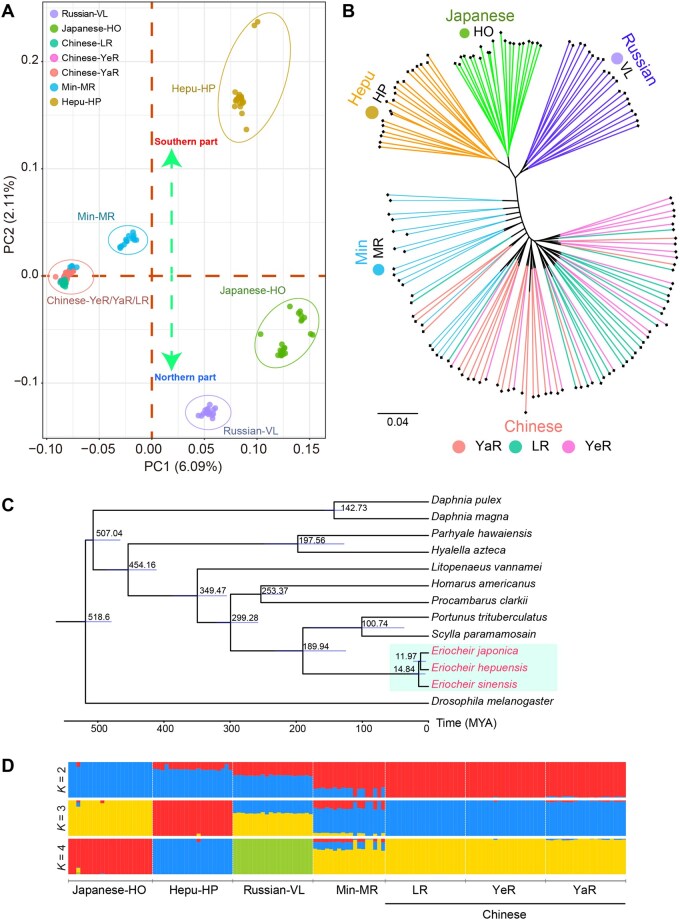
Population structure and evolutionary history across seven mitten crab populations **A**. PCA based on genome-wide SNPs. **B**. Phylogenetic tree constructed from high-quality SNPs, revealing evolutionary relationships among these populations. **C**. Phylogenetic tree constructed from one-to-one orthologs of 12 crustacean species. *Drosophila melanogaster* was used as the outgroup. Estimated divergence times are shown at the nodes. **D**. Population structure analysis of the different mitten crab populations under *K* values ranging from 2 to 4. PCA, principal component analysis; PC1/2, the first/second principal component; SNP, single-nucleotide polymorphism; MYA, million years ago.

Principal component analysis (PCA) of 912,659 SNPs (minor allele frequency > 0.05 and linkage disequilibrium pruned) showed clear genetic clustering among mitten crab populations. Four major clusters were observed, corresponding to the Chinese-YeR/YaR/LR, Japanese-HO, Hepu-HP, and Russian-VL populations. Additionally, the Min-MR population appeared in an intermediate position between the northern and southern clusters (**Figure 2**A). Meanwhile, a maximum-likelihood phylogenetic tree constructed from the whole-genome sequence data also confirmed the four clear clusters. The YaR, YeR, and LR populations clustered together, representing the Chinese mitten crab lineage. The Hepu-HP, Russian-VL, and Japanese-HO populations clustered together, representing the Japanese mitten crab lineage. The Min-MR population shows genetic similarities to the Chinese mitten crab, with several individuals clustering closely, suggesting potential admixture (Figure 2B).

In addition, comparative genomic analysis of 12 crustacean species with *Drosophila melanogaster* as an outgroup identified 684 one-to-one single-copy orthologous genes for phylogenetic assessment. The maximum-likelihood phylogenetic tree and molecular dating analysis revealed that *E. sinensis* and *E. japonica* represent distinct evolutionary lineages, diverging approximately 14.84 million years ago (MYA) (Figure 2C). This divergence supports their classification as separate species within the *Eriocheir* genus. Moreover, phylogeny analysis revealed a close clustering of *E. japonica* and *E. hepuensis*, indicating a strong phylogenetic relationship between these species (Figure 2C). Additionally, a phylogenetic tree based on mitochondrial genomes indicates a close relationship between *E. sinensis* and *E. hepuensis* (Figure S3A). The contrasting results from nuclear and mitochondrial genome analyses suggest that historical hybridization events may have influenced the genetic makeup of *E. hepuensis* (Figure S3A). Together, these findings confirm that *E. sinensis* and *E. japonica* are genetically distinct species, despite evidence of occasional genetic exchange in related populations.

Further, admixture analysis revealed distinct yet interconnected populations within the *Eriocheir* genus. At *K* = 2, there is a clear separation between the Chinese-YaR/YeR/LR and Japanese-HO mitten crab populations, with admixture signals detected in the Hepu-HP, Russian-VL, and Min-MR populations. At *K* = 3, the Hepu-HP mitten crab population aligns separately with Chinese-YaR/YeR/LR and Japanese-HO mitten crab populations, with admixture signals evident in the Russian-VL and Min-MR populations. At *K* = 4, further division shows that Min-MR is a hybrid with contributions from Chinese-YaR/YeR/LR, Japanese-HO, and Hepu-HP lineages (Figure 2D). Results from TreeMix analysis support these findings, indicating significant gene flow from the Chinese-YaR/YeR/LR mitten crab populations to the Hepu-HP, Russian-VL, and Min-MR populations, signifying extensive hybridization and introgression within the *Eriocheir* genus (Figure S3B). Pairwise sequentially Markovian coalescent (PSMC) analysis indicated small effective population size in Japanese-HO mitten crab (Figure S3C).

### Genomic differentiation and selection in the *Eriocheir* genus

**Figure 3:**
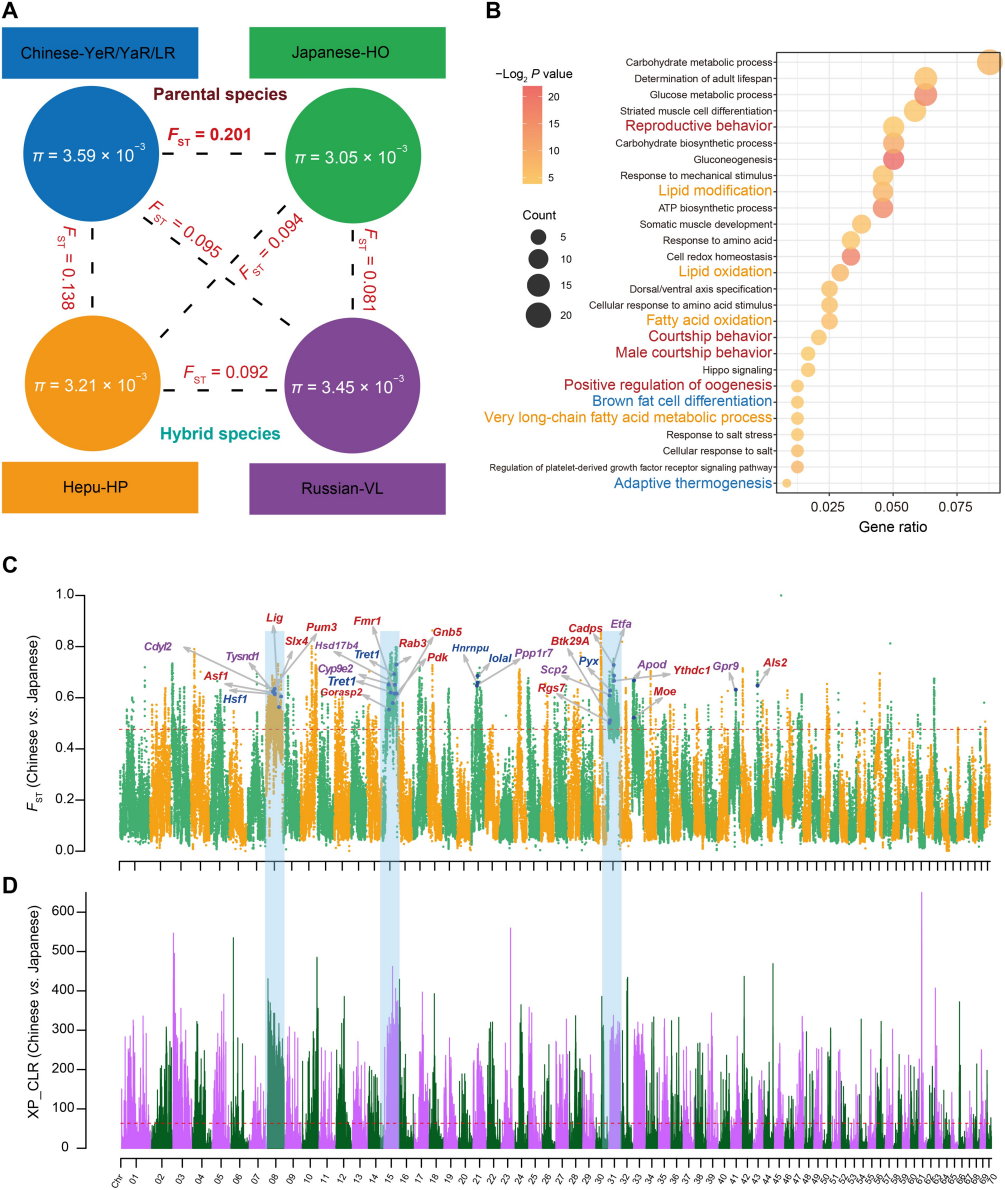
Genetic differentiation between Chinese and Japanese mitten crabs in the *Eriocheir* genus **A**. Nucleotide diversity (*π*) within population and genetic differentiation (*F*_ST_) between two populations. **B**. GO enrichment analysis of genes under divergent selection between Chinese and Japanese mitten crabs. GO terms related to reproduction, lipid oxidation, and temperature stimulus are colored in red, orange, and blue, respectively. Gene ratio denotes the proportion of differentially expressed genes that are annotated to a specific GO term relative to the total number of differentially expressed genes. **C**. Genomic-wide visualization of genetic differentiation (*F*_S__T_) between Chinese and Japanese mitten crabs. Genes linked to reproduction, lipid oxidation, and temperature stimulus are marked in red, purple, and blue, respectively. **D**. Genome-wide visualization of selection signals (XP-CLR scores) between Chinese and Japanese mitten crabs. In (C and D), selective regions with top 1% of *F*_ST_ values and XP-CLR scores are shaded in blue. GO, Gene Ontology; XP-CLR, Cross Population Composite Likelihood Ratio; Chr, chromosome.

Analysis of nucleotide diversity (*π*) among biallelic SNPs in mitten crab populations revealed substantial differences between the Chinese-YaR/YeR/LR and Japanese-HO lineages. The Chinese-YaR/YeR/LR populations exhibited the highest nucleotide diversity (*π* = 3.59 × 10^−3^), in contrast to the Japanese-HO population, which had the lowest *π* value of 3.05 × 10^−3^ (**Figure 3**A). This reduction in genetic diversity within the Japanese-HO population suggests a potentially restricted gene flow over time.

Whole-genome analyses of genetic differentiation further underscored the substantial evolutionary distance between the Chinese and Japanese mitten crab populations. The *F*_ST_ value, which measures population differentiation, revealed the largest genetic differentiation between the Chinese-YaR/YeR/LR and Japanese-HO populations, marked by a mean weighted *F*_ST_ value of 0.201 (Figure 3A). In contrast, the smallest genetic differentiation was observed among the three populations (LR, YeR, and YaR) of the Chinese mitten crab (Figure S4). Moreover, largest average nucleotide difference between populations (*D_xy_*) values were identified between Chinese-LR/YeR/YaR and Japanese-HO mitten crab populations (Table S8).

We identified notably differentiated genomic regions (top 1% of *F*_ST_ values) between the Chinese-YaR/YeR/LR and Japanese-HO mitten crabs, which contained a total of 401 genes (Table S9). Gene Ontology (GO) enrichment analysis indicated that these genes were enriched in lipid oxidation, muscle cell differentiation, determination of adult lifespan, adaptive thermogenesis, courtship behavior, and so on (Figure 3B). Regions with highly genomic differentiation were identified across almost entire chromosomes, such as Chr08, Chr15, and Chr31 (mean *F*_ST_ > 0.6), and these findings suggest that the Japanese-HO and Chinese-YaR/YeR/LR mitten crabs may have undergone divergent selection in these specific genomic regions after their speciation (Figure 3C and D). Functionally involved genes, such as *Asf1*, *Lig*, *Btk29A*, *Slx4*, and *Als2* associated with reproduction process, *Tysnd1*, *Cyp9e2*, *Hsd17b4*, and *Apod* associated with lipid oxidation, and *Hsf1*, *Tret1*, and *Pyx* associated with temperature stimulus, showed strong genetic differentiation and selection signals between Chinese and Japanese mitten crabs (Figure 3C and D).

Previous studies have identified the *Btk29A* gene, known for its role in courtship behavior and male genitalia development, as showing selection signals within the Japanese-HO mitten crab population [47,48]. Two distinct haplotypes of the *Btk29A* gene were identified in the Japanese-HO and Chinese-YaR/YeR/LR mitten crab populations. A G-to-A mutation on Chr31:3,522,309 led to alternative splicing in the *Btk29A* gene, causing the Japanese-HO mitten crab to lose exon 2 (Figure S5A and B). Furthermore, *Btk29A* exhibited significant differential expression between Chinese-YaR and Japanese-HO mitten crabs in the ovaries (Figure S5C). Additionally, the *Lingerer* (*Lig*) gene, known for its role in copulation initiation and termination [49], exhibited signs of divergent selection signals between the Japanese-HO and Chinese-YaR/YeR/LR mitten crab populations. A A-to-C mutation at position 1891 caused an amino acid substitution from methionine to leucine (Met631Leu), resulting in structural alterations in the lingerer protein (Figure S5D and E). Also, the expression of the *Lig* gene differed between the Chinese-YaR and Japanese-HO mitten crabs (Figure S5F). Moreover, the *Pyx* gene, responsible for cation channel activity linked to protection and tolerance against high-temperature stress [50], and the *Hsf1* gene, which plays a role in the heat shock response, displayed significant divergence, each with two distinct haplotypes identified between the Chinese-LR/YeR/YaR and Japanese-HO mitten crabs (Figure S6). Furthermore, the expression of both *Hsf1* and *Pyx* showed significant differences between Chinese-YaR and Japanese-HO mitten crabs (Figure S6). Notably, two missense mutations, c.1924 G>C in *Pyx* and c.732A>G in *Hsf1*, resulted in amino acid changes from aspartic acid to histidine (Asp642His) and from isoleucine to methionine (Ile244Met), respectively, in the Japanese-HO mitten crab (Figure S6). Additionally, Hepu-HP and Russian-VL mitten crabs, both of which are hybrids, exhibited a substantial proportion of heterozygous genotypes within their populations (Figure S6).

### Different genome architecture of the two hybrid mitten crabs

**Figure 4:**
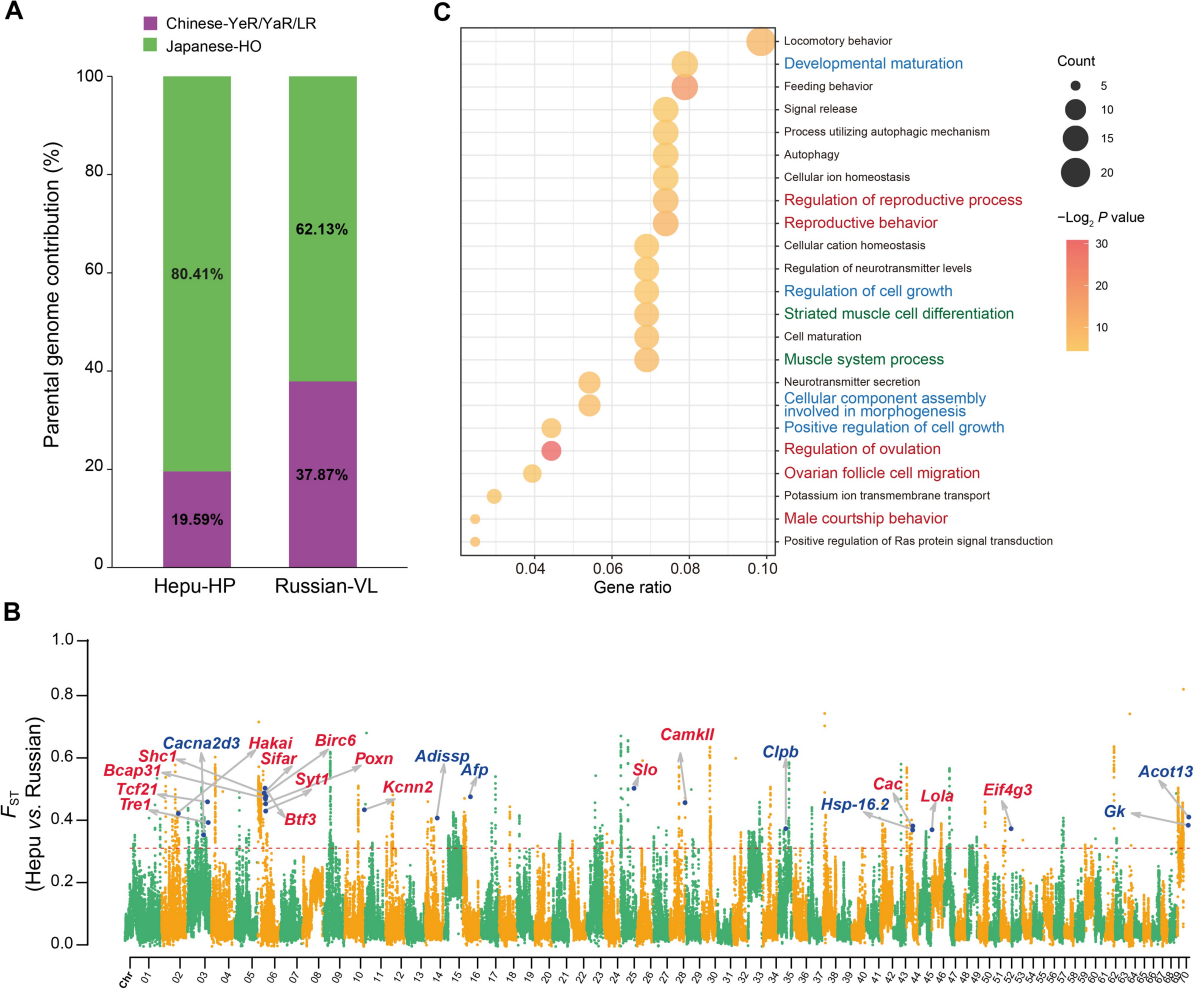
Different genome architectures between Hepu-HP and Russian-VL mitten crabs **A**. Bar chart illustrating parental genome contributions to the Hepu-HO and Russian-VL mitten crabs. **B**. Genome-wide visualization of genetic differentiation (*F*_ST_) between Hepu-HO and Russian-VL mitten crabs. Genes associated with reproduction and temperature stimulus are marked in red and blue, respectively. **C**. GO enrichment analysis of genes exhibiting genetic differentiation. GO terms related to reproduction, muscle development, and growth and development are colored in red, green, and blue, respectively. Gene ratio denotes the proportion of differentially expressed genes that are annotated to a specific GO term relative to the total number of differentially expressed genes.

Our results indicate that the Hepu-HP and Russian-VL mitten crabs have admixture ancestry (Figure 2). In both hybrid populations, there is a predominant genetic inheritance from the Japanese mitten crab, along with a lesser contribution from the Chinese mitten crab. In terms of their genome structure, Chinese and Japanese mitten crabs contribute about 19.59% and 80.41% to the genomic composition of Hepu mitten crabs, and 37.87% and 62.13% to that of Russian mitten crabs, respectively (**Figure 4**A). Interestingly, these two hybrid populations occupy different ecological environments at varying latitudes and display significant genomic differentiation across their entire genomes (Figure 4B). We identified 1734 highly differentiated genomic regions between the two hybrid populations (top 1% of *F*_S__T_ values), including 343 regions in Chr06 and 203 regions in Chr70, and no significantly differentiated regions were identified in Chr08 and Chr31 like their parental lineages, indicating that Hepu-HP and Russian-VL mitten crabs do not share differentiated regions with their parental species (Figure 4B). Furthermore, among the 390 genes identified in these regions, substantial genomic differentiation between the two hybrid populations was observed (Figure 4B and C; Table S10). GO enrichment analysis revealed that these genes were enriched in reproductive behavior, autophagy, muscle cell differentiation, regulation of ovulation, among others (Figure 4C). These findings suggest that hybrid populations may have experienced divergent selection following hybridization in distinct ecological environments.

The mean *F*_ST_ value was 0.138 between Hepu and Chinese mitten crabs, 0.094 between Hepu and Japanese mitten crabs, 0.095 between Russian and Chinese mitten crabs, and 0.081 between Russian and Japanese mitten crabs (Figure 3A, Figure S7). Moreover, the mean *F*_ST_ value between the two hybrid mitten crabs was 0.092 (Figure 3A). These findings suggest that both Hepu and Russian mitten crabs possess distinct genome architectures and are at an intermediate level of genetic differentiation (Figure 4B). Of the divergent SNPs (fixed) identified between the Chinese and Japanese mitten crabs, a notable majority (87.65% and 98.84%, respectively) were heterozygous in Hepu and Russian mitten crabs (Figure S8). Additionally, 1.17% and 0.46% of these SNPs matched the Chinese mitten crab genotype, while 11.18% and 0.70% matched the Japanese mitten crab genotype in Hepu and Russian mitten crabs, respectively (Figure S8).

### Natural selection in Hepu mitten crab

**Figure 5:**
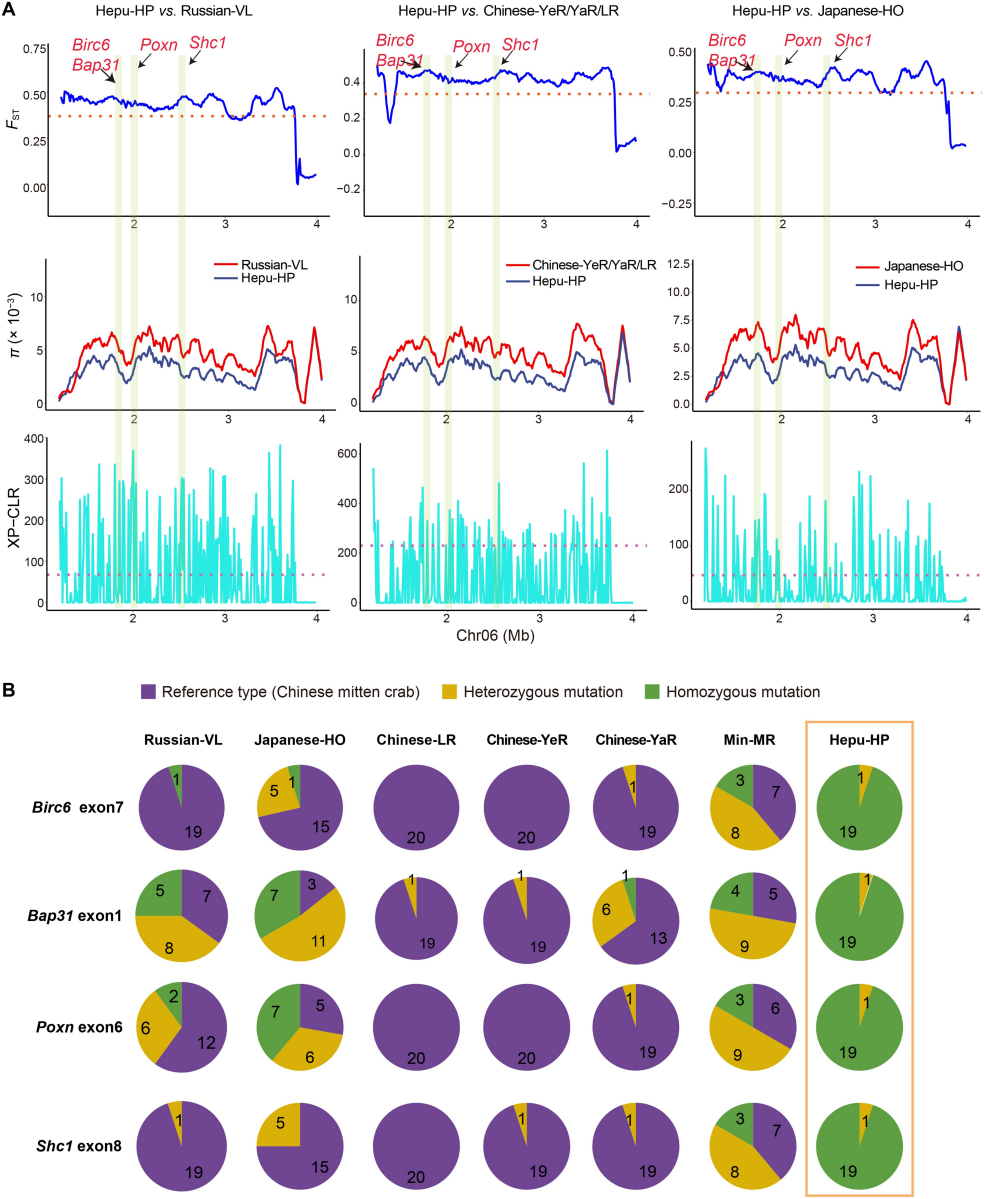
Detection of selection signals in Hepu-HP mitten crab **A**. Genomic comparisons of population differentiation (*F*_ST_), nucleotide diversity (*π*), and XP-CLR scores across mitten crab populations in specific regions of Chr06. Key loci (such as *Birc6*, *Bap31*, *Pxn*, and *Shc1*) showing selection signals in Hepu-HP mitten crab are highlighted. **B**. Pie charts showing the proportions of reference types (purple), heterozygous mutations (yellow), and homozygous mutations (green) across populations. Number within each pie chart indicates the number of individuals carrying the specific genotype in that population.

Remarkably, we detected strong selective sweep signals specific to the Hepu-HP mitten crab on Chr06 (**Figure 5**A). Within this region, several genes, including *Btf3*, *Birc6*, *Poxn*, *Syt1*, and *Shc1*, displayed clear signatures of natural selection. These selected genes are associated with the reproductive process and present nonsynonymous mutations in the Hepu mitten crab, indicating that these reproductive-related genes in Chr06 were selected by the natural environment (Figure 5A and B, Figure S9). Notably, the homozygous mutations were predominantly exclusive to the Hepu-HP mitten crab, while only reference allele genotypes were present in the Chinese mitten crab populations (LR, YeR, and YaR). Considering the Chinese mitten crab genotype as reference, the Japanese-HO mitten crab population exhibited only a small proportion of homozygous mutations (Figure 5B). The homozygous mutations observed in the Hepu mitten crab, likely influenced by strong selection, may have originated from the Japanese mitten crab through a hybridization event.

Moreover, we identified several temperature-response genes which displayed selection signals in the Hepu mitten crab, such as *Afp* and *Hsp-16.2*. Notably, nonsynonymous mutations were found in both genes: c.338 C>T in *Afp* and c.269 C>G in *Hsp-16.2*, resulting in amino acid changes from Pro to Leu and from Thr to Ser, respectively, in the Hepu mitten crab (Figure S10A and B). These genetic variations may have implications for temperature adaptation differences between the Hepu-HP mitten crab and Russian-VL mitten crab.

## Discussion

**Figure 6:**
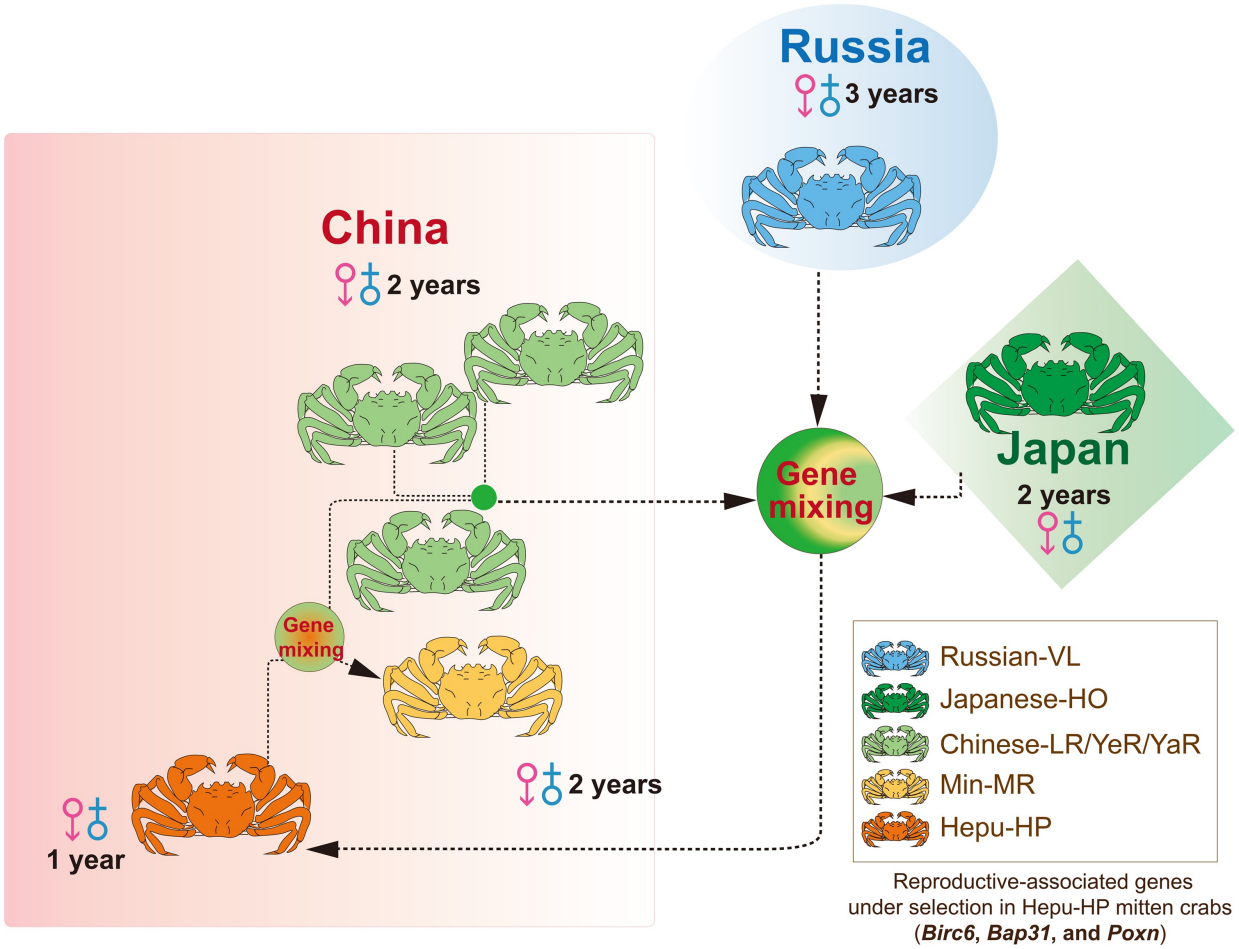
Hybridization and ecological distribution of the *Eriocheir* genus in East Asian This figure illustrates the genetic hybridization patterns and distribution of mitten crab populations across East Asia. The Hepu mitten crab exhibits genetic mixing with both Chinese and Japanese mitten crabs, acting as a hub of gene exchange. Additionally, the Min-MR mitten crab shows genetic mixing with both Hepu and Chinese mitten crabs, further contributing to regional genetic diversity. Arrows indicate the direction of gene flow, while colored circles represent areas of genetic mixing.

The unique central distribution of Chinese-LR/YeR/YaR and Japanese-HO mitten crabs has driven the formation of two ecologically significant hybrid populations, Russian-VL mitten crab to the north and Hepu-HP mitten crab to the south, demonstrating how geographical proximity and genetic intermixing foster adaptive diversity within the *Eriocheir* genus (**Figure 6**) [45]. Genetic and phenotypic analyses suggest that the Hepu-HP mitten crab has diverged significantly, potentially representing a distinct species within the *Eriocheir* genus. Through our research, we have illuminated the intricate history of hybridization within the *Eriocheir* genus. This discovery resolves long-standing taxonomic debates that have spanned decades [30,31,33].

### Multilayered hybridization history of the *Eriocheir* genus

Chinese-YeR, Chinese-YaR, Chinese-LR, Japanese-HO, Russian-VL, and Hepu-HP mitten crab populations show distinct geographic, morphological, evolutionary, and genetic differences. Hepu-HP and Russian-VL mitten crabs originated from hybridization between Chinese and Japanese mitten crabs, followed by divergent selection that led to distinctive phenotypic traits (Figures 4 and 6), illustrating how hybridization and selection drive diversity within the *Eriocheir* genus. What is even more intriguing is that mitten crabs collected from the Min River region exhibit genetic admixture signals, signifying their hybrid nature. These mitten crabs display a complex admixture ancestry with genetic contributions from the Chinese, Japanese, and Hepu mitten crabs, reflecting their intermediate geographic position (Figures 2D and 6, Figure S3B). This pattern suggests that hybridization in the Min-MR mitten crab likely occurred after the formation of the Hepu mitten crab, pointing to a more recent hybridization event involving the Chinese and Hepu mitten crabs [34,35]. In light of the intricate and recurring hybridization within the *Eriocheir* genus, it is reasonable to suspect that the invasive mitten crabs in Europe and North America may likewise be of hybrid origin, as earlier studies have proposed [44]. Confirming this hypothesis will necessitate additional research in the future.

Prior research has suggested that the Hepu mitten crab should be classified as an independent clade rather than a hybrid. This distinction can be attributed to two key factors: (1) the limited discriminatory power of utilizing only Amplified Fragment Length Polymorphism (AFLP) loci and Cytochrome C Oxidase Subunit II (COII) markers, and (2) the case that the Hepu mitten crab is an ancient hybrid that has undergone adaptive evolution which will form unique genome architecture [51]. In this study, Hepu-HP mitten crab shows a high level of heterozygosity, likely due to its hybrid origin involving Chinese and Japanese mitten crabs (Figure S8).

Previous reports suggest that Japan island separation from the southeastern margin of the Chinese continent 15–25 MYA, driven by crustal movements [52,53], may contribute to the speciation event of the Chinese and Japanese mitten crabs. Mitten crabs are catadromous species that require seawater for reproduction. The isolation of the Japan sea from China’s marginal seas such as the Yellow sea and the East sea, likely contributed to reproductive isolation (RI) and differentiation. This is because mitten crabs, which are offshore spawners, cannot migrate to the deep sea to reproduce, and they die after reproduction [35,51,54]. The divergence of the Chinese and Japanese mitten crabs was estimated at around 14.84 MYA, which aligns with the formation time of the Japan island (Figure 2C).

### Adaptive evolutionary path of Hepu-HP and Russian-VL mitten crabs through hybridization

The differences in morphological traits mirror the process of adaptive evolution, as seen in the unique ecotype phenotypes of the Russian-VL and Hepu-HP mitten crabs. These distinct characteristics provide potential evidence of their adaptations to diverse environmental conditions [45]. Diverse environments may impose strong selection pressure that has shaped their distinct phenotypes (Figure 1, Figure S1). Genomic analysis provides evidence that the Russian-VL and Hepu-HP mitten crabs have undergone divergent selection, with specific genes related to their adaptation being selected (Figure 4). The observed differences in reproductive maturity and morphology between mitten crabs from northern and southern regions highlight how environmental factors shape species adaptations. Specifically, Hepu-HP mitten crabs, located in the warmer, southern region of China, mature within a year. In contrast, Russian-VL mitten crabs, residing in colder, northern regions, take 3 years to reach maturity [55]. Analysis of Hepu mitten crab identified strong selection signals in genes related to their reproduction and temperature adaptation (Figure 5). The accelerated reproductive maturity in Hepu-HP mitten crabs may be an adaptive response to their warmer environment, where the annual mean temperature exceeds 20°C — the reported threshold for optimal mitten crab growth and development [56]. This elevated temperature may impose strong selection on genes associated with reproductive timing and temperature resilience in the Hepu-HP mitten crab population, contributing to their distinct reproductive strategies and morphology (Figure S2). We also observed that the body weight of the Russian-VL mitten crab was higher than that of the Hepu-HP mitten crab (Figure S1), suggesting that environmental factors may play a role. A similar phenomenon was noted in mice, where northern population had significantly larger sizes than those closer to the equator [57]. It is possible that Hepu (Guangxi Zhuang Autonomous Region, China) might not be the ideal habitat for mitten crabs. Nevertheless, hybridization could play a role in expanding the distribution of Hepu mitten crabs to southern areas, resulting in smaller body sizes, improved heat tolerance, and varied sexual maturity times for better adaptation (Figure 6).

### Evidence for a hybrid speciation event

Despite naming his book “On the Origin of Species”, Darwin provided a sketchy blueprint for how species form in nature, referring to it as the “mystery of mysteries” [58]. A key part of hybrid speciation is how hybrids separate from their parental species by developing RI. This helps them keep their own genetic identity and evolve into a new species [22,59,60]. Research indicated that the reproduction season and sexual maturity time (1 year) of Hepu-HP mitten crab are different from their parents [61]. In our research, we identified reproductive-associated genes such as *Birc6*, *Bap31*, *Poxn*, *Syt1*, and *Shic1* which are involved in oogenesis, spermatogenesis, and courtship behavior. These genes showed strong selection signals in Hepu-HP mitten crab, suggesting that they may contribute to the development of RI between the Hepu mitten crab and its parental species (Figure 5). However, the molecular functions of these selected genes need to be verified in future studies. The Hepu-HP mitten crab is known for its abbreviated sexual maturity period, smaller body size, and its unique habitat (the only mitten crab species living in the southernmost region with higher annual temperature) (Figure S2). These factors account for the significant ecological differences observed between the Hepu-HP mitten crab and its parental species [28]. In the culture process of the Chinese mitten crab, a high temperature is detrimental to survival and will promote the precocity, consistent with the characteristics of the Hepu mitten crab [61]. Together, our results pointed out large genetic differentiation, significant phenotype variation, and established RI between the Hepu mitten crab and its parental species, indicating that the Hepu mitten crab is an independent ecological species formed through homoploid hybrid speciation event [22].

## Conclusion

In conclusion, our study unravels a complex tapestry of hybridization within the *Eriocheir* genus. Notably, the Hepu-HP and Russian-VL mitten crabs, as key hybrid species, exhibit substantial genomic differentiation and have experienced adaptive evolution (Figure 6). To unlock the secrets of mitten crab adaptation, future research should investigate additional populations, including those in invasive regions, and explore the molecular functions of the identified genes that have been selected by different environments. These intricate hybridization events have likely played a significant role in shaping species diversity within the *Eriocheir* genus and have had a significant impact on their global distribution patterns.

## Materials and methods

### Sample collection and morphological characterization

A total of 139 individuals from 7 populations (LR, YeR, YaR, VL, HO, MR, and HP) of *E. sinensis*, *E. japonica*, *E. hepuensis*, and their hybrid populations were collected from 2015 to 2017 (Table S5). The geographical locations of all collected mitten crab individuals were recorded with a global positioning system on the WGS84 datum (World Geodetic System 1984). All the collected adult mitten crab samples were measured for body weight (BW), shell length (L), shell width (A6), length of frontal teeth (A1–A5), and limb length (F1 and F2). All morphological measurements except body weight (BW) were normalized by dividing by the shell length (L). LDA was conducted using the normalized morphological data by machine learning methods, and comparisons among the seven populations for all morphological measurements were conducted by ANOVA. Temperature data from 1960 to 2020 were obtained from WorldClim (v2.1), a high-resolution climate database accessible at https://www.worldclim.org/data/worldclim21.html [62]. This database provides global, monthly temperature records at various spatial resolutions, supporting robust analysis of long-term climate trends.

### Genome assembly and gene annotation of *E. japonica* and *E. hepuensis*

For *E. japonica*, an adult wild male individual from the Hokkaido was collected. Pair-end libraries of 180 bp, 500 bp, and 800 bp, as well as mate-pair-end libraries of 2 kb, 5 kb, and 10 kb, were constructed and sequenced on the Illumina HiSeq 2500 platform (2 × 150 bp). Additionally, a 20-kb DNA library was created and sequenced on PacBio RS II platform. Furthermore, a 40-kb DNA library was constructed following the 10X Genomics sequencing library construction pipeline and sequenced on Illumina HiSeq 4000 platform. Initially, clean reads were assembled into contigs using Platanus (v2.0.2) [63] and subsequently linked via Redundans with default parameters [64]. Gaps were closed utilizing PBJelly2 with PacBio raw reads, followed by error correction [65]. Rascaf (v1.0.2) was employed to further link the assemblies using transcriptome sequencing data [66]. Finally, scaffolds were further linked using 10X Genomics linking reads with Assembly Round-up by Chromium Scaffolding (ARCS; v1.2.1) [67] and Long Interval Nucleotide K-mer Scaffolder (LINKS; v2.0) [68].

For *E. hepuensis*, an adult wild male individual was collected. Pair-end libraries of 250 bp, 400 bp, and 800 bp, along with mate-pair-end libraries of 2 kb, 5 kb, and 10 kb, were constructed. These libraries were sequenced on an Illumina HiSeq 4000 platform. Following the filtration of low-quality reads, all clean reads were assembled into contigs using SOAPdenovo2 with *K* = 27, 31, and 99 [69]. The resulting contigs were further linked into scaffolds using Redundans with default parameters [64]. To refine the assemblies, Rascaf (v1.0.2) was utilized for further linkage of the mitochondrial-genome-free assemblies, employing transcriptome sequencing data.

Five organs (eyestalk, gill, hepatopancreas, muscle, and ovary) were collected from *E. japonica* and *E. hepuensis* and used for RNA-seq. The RNA-seq libraries were constructed with Truseq RNA Sample Prep Kit for Illumina (Catalog No. RS-122-2001, Illumina, San Diego, CA) and sequenced on the Illumina HiSeq 4000 platform with pair-end mode (2 × 150 bp). The RNA-seq data were used for genome assembly and annotation.

Transcriptome alignment, *de novo* gene prediction, and sequence homology-based predictions implemented in EVidenceModeler (EVM) were used for gene prediction [70]. Initially, RNA-seq reads were subjected to transcriptome alignment and assembled into transcripts using Spliced Transcripts Alignment to a Reference (STAR; 2.7.10a) and StringTie (v2.2.0) [71,72]. These transcripts were then aligned to the genomes to acquire gene structure annotation information through Program to Assemble Spliced Alignments (PASA; v2.5.1) [73]. For *de novo* gene prediction, GeneMark-ET and AUGUSTUS were used to predict genes on transposable-elements-hard-masked genome sequences [74,75]. A high-quality dataset was generated by PASA (v2.5.1) to train these *ab initio* gene predictors. Additionally, sequence homology-based gene prediction incorporated protein sequences from the SwissProt vertebrate database and six organisms (*E. sinensis*, *Daphnia pulex*, *Lepeophtheirus salmonis*, *Caenorhabditis elegans*, *D. melanogaster*, and *Homo sapiens*) into Gene Model Mapper (GeMoMa; v1.9) to generate homology gene structures. Subsequently, all predicted gene structures were integrated into consensus gene models using EVM (v2.0.0) [70]. To determine the functional annotation of the gene models, a Basic Local Alignment Search Tool for Proteins (BLASTP) search was conducted against public protein databases, including non-redundant protein sequences (NR) in National Center for Biotechnology Information (NCBI), SwissProt, RefSeq, and Kyoto Encyclopedia of Genes and Genomes (KEGG) with an E-value threshold of ≤ 1E−5. Additionally, the motifs and domains of each gene model were predicted using InterProScan 5 against public protein databases, including Protein Domain Families (ProDom), Protein Motif Fingerprints (PRINTS), Protein families (Pfam), Gene 3-Dimensional (Gene3D), Conserved Domain Database (CCD), Simple Modular Architecture Research Tool (SMART), Protein Analysis Through Evolutionary Relationships (PANTHER), Protein Site (PROSITE), and Superfamily Database of Structural and Functional Annotation (SUPERFAMILY) [76].

### Whole-genome resequencing library construction and resequencing

In each mitten crab population, 18–21 individuals were selected for DNA extraction and sequencing library construction. DNA was extracted from muscle tissue using the QIAGEN DNeasy Blood & Tissue kit (Catalog No. 69504, QIAGEN, Shanghai, China). The concentration and purity of extracted DNA were measured by NanoDrop 2000. After quality control, DNA sequencing libraries with 350–400 bp insert size were sequenced on the Illumina NovaSeq 6000 platform with paired-end mode (150 bp read length).

### Genome read mapping and variant calling

Raw reads were filtered by Trimmomatic (v0.39) using default parameters [77]. After filtering, all the clean reads from each individual were aligned to the *E. sinensis* reference genome using Burrows–Wheeler Aligner (BWA; v0.7.17) (parameter: -t 12 -M -R) [78]. The aligned Binary Alignment/Map format (BAM) files were then sorted using SAMtools (v1.7), and polymerase chain reaction (PCR) duplicates were marked by MarkDuplicates module by Genome Analysis Toolkit (GATK; v4.3.0.0) [79]. Then, the GATK HaplotypeCaller module was run on each BAM file to generate genomic variant call format (GVCF) files. The GVCF files from the 139 individuals were combined into a single GVCF file by CombineGVCFs implemented in GATK (v4.3.0.0), and then the variant call format (VCF) files were obtained through GenotypeGVCFs command. The SNPs were further hard filtered using the following parameters: (1) QD < 2.0 ‖ MQ < 40.0 ‖ FS > 60.0 ‖ SOR > 3.0 ‖ MQRankSum < −12.5 ‖ ReadPosRankSum < −8.0, (2) read depths < 2, and (3) only biallelic were kept. The SNPs were annotated by SnpEff (v5.0) [80]. SNPs were classified as synonymous, nonsynonymous, intron, intergenic region, and so on (Table S7).

### Population structure analysis

For the population structure and phylogenetic analysis, variants with missing rate > 20% and minor allele frequency < 0.05 were removed using PLINK (v1.9) [81]. Meanwhile, to avoid linkage disequilibrium which will affect the population structure, all loci within 50-kb windows with an *r*^2^ exceeding 0.2 (--indep-pairwise 50 10 0.2) were filtered. The phylogenetic tree was constructed using IQ-TREE (v2.0.3; parameters: -bb 1000 -mem 120G -nt 16 -m MFP+ASC -st DNA) with 1000 bootstrap replicates [82]. The population structure analysis was conducted using ADMIXTURE (v2.34) with 30,000 burning and 20,000 interactions [83]. PCA was conducted by smartpca (v18140) using filtered biallelic SNPs. Gene flow was detected with TreeMix (v1.13), which infers a maximum-likelihood tree based on genome-wide allele frequency data [84]. Demographic histories of the mitten crabs were reconstructed using the PSMC model with the mutation rate (μ) set to 7 × 10^−9^, and the generation time (g) set to 2 years [85].

### Genome scans for natural selection

We used VCFtools (v0.1.17) to calculate the pairwise *F*_ST_ among the seven mitten crab populations with biallelic SNPs [86] and the nucleotide diversity (*π*) for each population. Both metrics were computed using a window size of 100 kb and a step size of 10 kb. Windows ranked in the top 1% of both *F*_ST_ values and *π* ratios were considered putative selective regions. Selective sweeps were also detected using the Cross Population Composite Likelihood Ratio (XP-CLR) method. Each chromosome was analyzed with the following parameters: --rrate 3.09e-8 --ld 0.95 --maxsnps 200 --size 100000 --step 10000 [87]. Windows in the top 1% of XP-CLR scores were considered candidate selective regions.

### Mitochondrial genome assembly and phylogenetic tree construction

The mitochondrial genome was *de novo* assembled by NOVOPlasty using the whole-genome sequencing data from the individuals of the seven populations with the mitochondrial genome sequence of *E. sinensis* as reference [88]. After assembly, the mitochondrial genome sequences were manually inspected, and five representative sequences from each population were selected for phylogenetic tree construction using IQ-TREE with 1000 bootstrap replicates [82].

### Species tree construction and divergence time estimation

Protein sequences of *D. pulex*, *Daphnia magna*, *Parhyale hawaiensis*, *Hyalella azteca*, *Litopenaeus vannamei*, *Homarus americanus*, *Procambarus clarkii*, *Portunus trituberculatus*, *Scylla paramamosain*, *E. sinensis*, and *D. melanogaster* were downloaded from the NCBI database except for *E. japonica* and *E. hepuensis*. Species trees were constructed by IQ-TREE using the 684 one-to-one single-copy orthologous genes identified by OrthoFinder (v2.3.11) [89]. We further employed Markov Chain Monte Carlo Tree (MCMCTree) in PAML (v4.8) [90] to estimate the divergence time of the species using the calibration time as a constraint, including *D. melanogaster–D. pulex* (474.8–530.0 MYA), *H. americanus–P. clarkii* (241.0–321.6 MYA), and *D. magna–D. pulex* (130–158 MYA), which were derived from the TimeTree database [91]. The MCMCTree was run for 500,000 iterations, and the first 50,000 samples were burned in. We ran the program three times to confirm that the results were similar between runs.

## Ethical statement

The sampling procedures and experimental protocols in this study were approved by the Institutional Animal Care and Use Committee of Shanghai Ocean University (Shanghai, China) on the care and use of animals for scientific purposes (Approval No. SHOU-DW-2023-033).

## Supplementary Material

qzaf079_Supplementary_Data

## Data Availability

The raw resequencing data of the seven populations reported in this study have been deposited in the Genome Sequence Archive (GSA) [92] at the National Genomics Data Center (NGDC), China National Center for Bioinformation (CNCB) (GSA: CRA013348), and are publicly accessible at https://ngdc.cncb.ac.cn/gsa. The genome assemblies of *E. japonica* and *E. hepuensis* reported in this study have been deposited in the Genome Warehouse [93] at the NGDC, CNCB (GWH: GWHFIFC00000000.1 and GWHFIFD00000000.1), and are publicly accessible at https://ngdc.cncb.ac.cn/gwh. The RNA-seq data of *E. japonica* and *E. hepuensis* reported in this study have been deposited in the GSA [92] at the NGDC, CNCB (GSA: CRA020935), and are publicly accessible at https://ngdc.cncb.ac.cn/gsa.

## CRediT author statement

**Jun Wang:** Conceptualization, Methodology, Data analysis, Visualization, Writing – original draft, Writing – review & editing. **Xin Hou:** Data curation, Visualization. **Xiaowen Chen:** Methodology, Data curation. **Roland Nathan Mandal**: Data analysis, Visualization. **Nusrat Hasan Kanika:** Visualization, Writing – original draft, Writing – review & editing. **Chunhong Yuan:** Resources, Writing – review & editing. **Yongju Luo:** Resources, Writing – review & editing. **Chenghui Wang:** Conceptualization, Methodology, Supervision, Writing – original draft, Writing – review & editing. All authors have read and approved the final manuscript.
