# Supplementary material for: Genomic Insights into Hybridization and Speciation of Mitten Crabs in the Eriocheir Genus
Source: Genomics Proteomics Bioinformatics. 2025 Sep 15;23(6):qzaf079. doi: 10.1093/gpbjnl/qzaf079 (PMC12996911; doi:10.1093/gpbjnl/qzaf079)
Supplement: qzaf079_Supplementary_Data [file qzaf079_supplementary_data.zip › Figure S1.pdf]

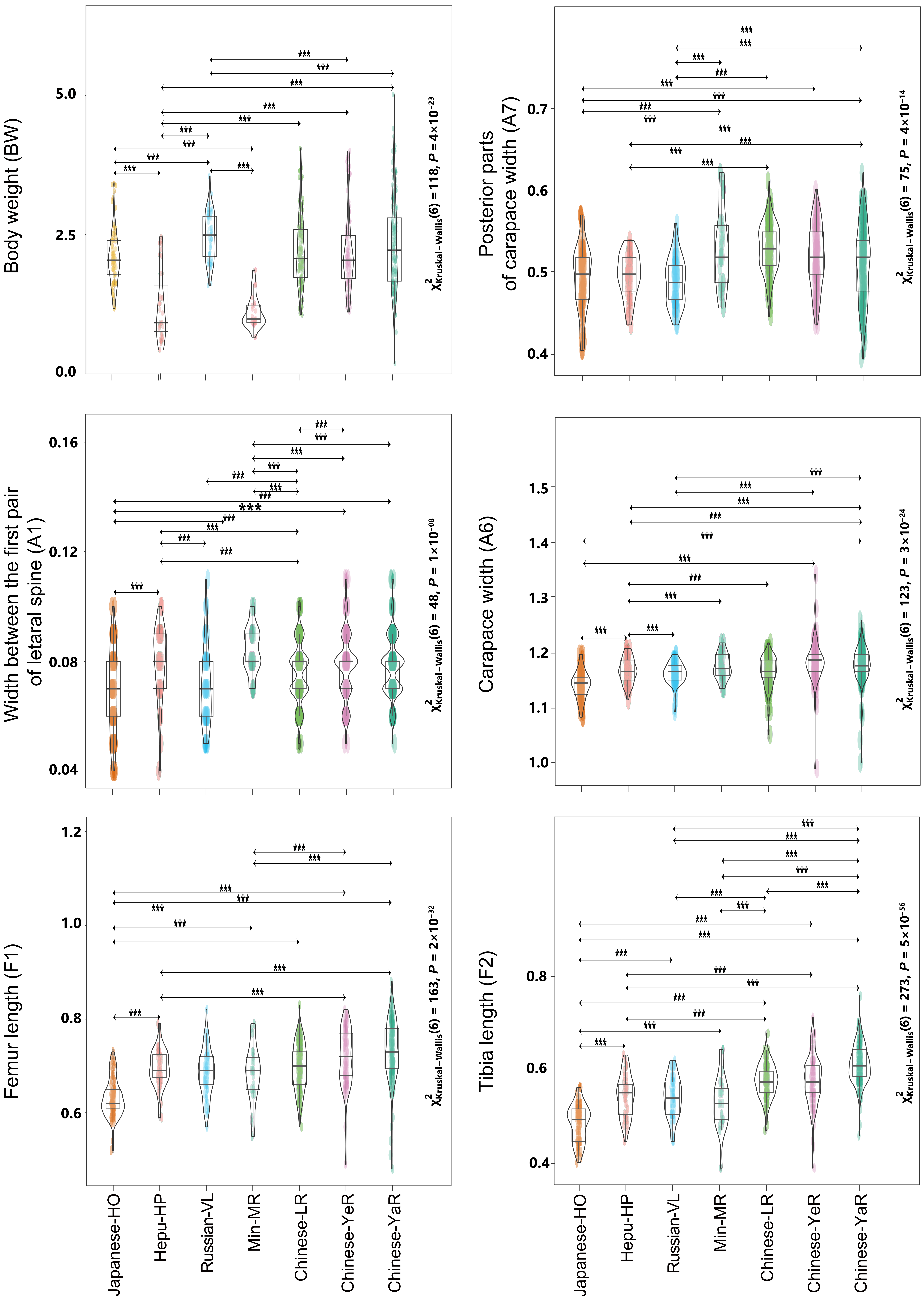

Body weight (BW)
$\chi^2_{Kruskal-Wallis}(6) = 118, P = 4\times10^{-23}$
Posterior parts of carapace width (A7)
$\chi^2_{Kruskal-Wallis}(6) = 75, P = 4\times10^{-14}$
Width between the first pair of letaral spine (A1)
$\chi^2_{Kruskal-Wallis}(6) = 48, P = 1\times10^{-08}$
Carapace width (A6)
$\chi^2_{Kruskal-Wallis}(6) = 123, P = 3\times10^{-24}$
Femur length (F1)
$\chi^2_{Kruskal-Wallis}(6) = 163, P = 2\times10^{-32}$
Tibia length (F2)
$\chi^2_{Kruskal-Wallis}(6) = 273, P = 5\times10^{-56}$
Japanese-HO
Hepu-HP
Russian-VL
Min-MR
Chinese-LR
Chinese-YeR
Chinese-YaR
