# Supplementary material for: Genomic Insights into Hybridization and Speciation of Mitten Crabs in the Eriocheir Genus
Source: Genomics Proteomics Bioinformatics. 2025 Sep 15;23(6):qzaf079. doi: 10.1093/gpbjnl/qzaf079 (PMC12996911; doi:10.1093/gpbjnl/qzaf079)
Supplement: qzaf079_Supplementary_Data [file qzaf079_supplementary_data.zip › Supplementary material captions.docx]

**Supplementary material**

**Figure S1 Comparative analysis of morphological traits in seven mitten crab populations using ANOVA**

Violin plots display the variation in phenotypic traits (A1, A6, A7, F1, and F2) and BW across mitten crab populations from Japanese-HO, Hepu-HP, Russian-VL, Min-MR, Chinese-YeR/YaR/LR. For consistency, all morphological traits (A1, A6, A7, F1, and F2) were normalized by shell length (L), with the exception of BW. Significant differences between groups are indicated by *** (*P* < 0.001). Refer to Figure 1C for trait definitions. BW, body weight; ANOVA, analysis of variance.

**Figure S2 Reproductive maturity seasons across mitten crab populations from varied geographic regions**

**A.** Ovary and testis characteristics of mitten crab populations: Russian mitten crab (a, e), Chinese mitten crab (b, f), Japanese mitten crab (c, g), and Hepu mitten crab (d, h). **B.** Graphs illustrate temperature changes in physiological indices across different geographic populations.

**Figure S3 Mitochondrial genome, TreeMix, and demographic history analysis of mitten crab populations**

**A.** Phylogenetic tree of mitochondrial genomes in mitten crabs from the 7 populations, with *Xenograpsus testudinatus* (NC_013480) as an outgroup. **B.** TreeMix analysis displaying inferred migration edges among those populations. **C.** Demographic history of mitten crabs from the 7 populations.

**Figure S4 Genetic diversity and genetic differentiation values among Chinese mitten crab populations**

**Figure S5 Divergent selection between Chinese and Japanese mitten crab populations, based on *Btk29A* and *Lig* gene**

**A.** Detection of selection signals in the *Btk29A* gene, with a comparison of gene structure between Chinese and Japanese mitten crabs. **B.** Haplotype diversity of the *Btk29A* gene across different mitten crab populations. **C.** *Btk29A* gene expression in the ovary, demonstrating significant differential expression between Chinese-YaR and Japanese-HO mitten crabs (*P* < 0.01). **D.** Identification of selection signals in the *Lig* gene. **E.** Sequence alignment of partial Lig proteins, highlighting mutations. **F.** *Lig* gene expression in the ovary, showing distinct expression patterns between Chinese-YaR and Japanese-HO mitten crabs. TPM, Transcripts Per Kilobase of exon model per Million mapped reads.

**Figure S6 Geographic variation and genetic divergence in *Hsf1* and *Pyx* genes of Chinese and Japanese mitten crabs**

The two circles indicate the haplotype of *Hsf1* (left circle) and *Pyx* (right circle) gene. Number in the circle indicates the number of individuals in this population with the specific genotype. The expression value (TPM) of *Hsf1* and *Pyx* gene in the ovary of Chinese and Japanese mitten crab were present in the right corner. The temperature data were downloaded from the WorldClim 2 website (https://www.worldclim.org/data/worldclim21.html).

**Figure S7 Genome-wide genetic differentiation (*Fst* value) between mitten crab populations**

**A.** Genetic differentiation value between Hepu mitten crab and Chinese mitten crab. **B.** Genetic differentiation value between Hepu mitten crab and Japanese mitten crabs. **C.** Genetic differentiation value between Russian mitten crab and Chinese mitten crab. **D.** Genetic differentiation value between Russian mitten crab and Japanese mitten crabs.

**Figure S8**  **Frequency of divergent allele genotypes from Chinese-YeR/YaR/LR, and Japanese-HO mitten crabs in Hepu-HP and Russian-VL mitten crabs**

**Figure S9 Haplotype figures of *Birc6, Bap31, Poxn, and Shc1* genes among the selected populations of mitten crab**

**Figure S10** **Genetic and structural analysis of *Afp* and *Hsp-16.2* genes from different mitten crab populations**

**A.** Sequence alignment, protein structure, and haplotype frequency of the *Afp* gene. **B.** Sequence alignment, protein structure, and haplotype frequency of the *Hsp-16.2* gene.

**Table S1 Raw sequencing data information for *Eriocheir japonica* genome assembly across multiple sequencing platforms**

**Table S2 Raw sequencing data information for *Eriocheir hepuensis* genome assembly**

**Table S3 Genome assembly metrics of three mitten crab species: *Eriocheir sinensis*, *Eriocheir japonica*, and *Eriocheir hepuensis***

**Table S4** **Comparison of BUSCO assessment results among *Eriocheir* genomes**

**Table S5 Information of sampling localities, and sample size of the 7 populations of *Eriocheir* species**

**Table S6 Sequencing and mapping information of the 139 mitten crab individuals from the 7 populations of *Eriocheir* species**

**Table S7 SNPs annotation information by SnpEff software**

**Table S8 Pairwise *Dxy* values identified among different *Eriocheir* populations**

**Table S9 Functional annotation of the 401 genes that showed highest *Fst* value (Top 1%) between the Chinese-LR/YeR/YaR, and Japanese-HO mitten crab**

**Table S10 Functional annotation of the 390 genes that showed highest *Fst* value (Top 1%) between the Hepu-HP and Russian-VL mitten crab**
