# Supplementary material for: Genomic Insights into Hybridization and Speciation of Mitten Crabs in the Eriocheir Genus
Source: Genomics Proteomics Bioinformatics. 2025 Sep 15;23(6):qzaf079. doi: 10.1093/gpbjnl/qzaf079 (PMC12996911; doi:10.1093/gpbjnl/qzaf079)
Supplement: qzaf079_Supplementary_Data [file qzaf079_supplementary_data.zip › Table S1.docx]

**Table S1 Raw sequencing data information for *Eriocheir japonica* genome assembly across multiple sequencing platforms**

| Insert size | Sequencing platform | Mode | No. of reads | No. of bases | Sequencing depth (×) |
| --- | --- | --- | --- | --- | --- |
| 180 bp | Illumina HiSeq 2500 | PE125 | 509,344,202 | 64,177,369,452 | 41.95 |
| 500 bp | Illumina HiSeq 2500 | PE125 | 445,488,362 | 56,131,533,612 | 36.69 |
| 800 bp | Illumina HiSeq 2500 | PE125 | 422,638,190 | 52,829,773,750 | 34.53 |
| 2 kb | Illumina HiSeq 2500 | PE125 | 503,451,454 | 63,434,883,204 | 41.46 |
| 5 kb | Illumina HiSeq 2500 | PE125 | 317,390,542 | 39,991,208,292 | 26.14 |
| 10 kb | Illumina HiSeq 2500 | PE125 | 126,462,706 | 15,934,300,956 | 10.41 |
| 10 kb | Illumina HiSeq 4000 | PE150 | 224,930,908 | 33,739,636,200 | 22.05 |
| Total | - | - | 2,549,706,364 | 326,238,705,466 | 213.23 |
|  | PacBio platform | - | 3,559,898 | 21,799,025,296 | 14.25 |
|  | 10X Genomics platform | PE150 | 784,212,882 | 117,631,932,300 | 76.88 |

*Note*: Raw reads data were generated for the *Eriocheir japonica* genome sequencing project using Illumina platform, PacBio platform, and 10X Genomics platforms.
