# Supplementary material for: Genomic Insights into Hybridization and Speciation of Mitten Crabs in the Eriocheir Genus
Source: Genomics Proteomics Bioinformatics. 2025 Sep 15;23(6):qzaf079. doi: 10.1093/gpbjnl/qzaf079 (PMC12996911; doi:10.1093/gpbjnl/qzaf079)
Supplement: qzaf079_Supplementary_Data [file qzaf079_supplementary_data.zip › Table S2.docx]

**Table S2 Raw sequencing data information for *Eriocheir hepuensis* genome assembly**

| Insert size | Sequencing platform | Mode | No. of reads | No. of bases | Sequencing depth (×) |
| --- | --- | --- | --- | --- | --- |
| 250 bp | Illumina HiSeq 4000 | PE150 | 823,719,318 | 123,557,897,700 | 76.27 |
| 400 bp | Illumina HiSeq 4000 | PE150 | 736,711,770 | 110,506,765,500 | 68.21 |
| 800 bp | Illumina HiSeq 4000 | PE150 | 458,664,708 | 57,333,088,500 | 35.39 |
| 2 kb | Illumina HiSeq 4000 | PE150 | 766,300,218 | 114,945,032,700 | 70.95 |
| 5 kb | Illumina HiSeq 4000 | PE150 | 466,914,348 | 70,037,152,200 | 43.23 |
| 10 kb | Illumina HiSeq 4000 | PE150 | 355,560,222 | 53,334,033,300 | 32.92 |
| Total |  |  | 3,607,870,58 | 529,713,969,900 | 326.98 |
