# Supplementary material for: Genomic Insights into Hybridization and Speciation of Mitten Crabs in the Eriocheir Genus
Source: Genomics Proteomics Bioinformatics. 2025 Sep 15;23(6):qzaf079. doi: 10.1093/gpbjnl/qzaf079 (PMC12996911; doi:10.1093/gpbjnl/qzaf079)
Supplement: qzaf079_Supplementary_Data [file qzaf079_supplementary_data.zip › Table S3.docx]

**Table S3 Genome assembly metrics of three mitten crab species: *Eriocheir sinensis*, *Eriocheir japonica*, and *Eriocheir hepuensis***

|  | ***Eriocheir sinensis**** | ***Eriocheir japonica*** | ***Eriocheir hepuensis*** |
| --- | --- | --- | --- |
| Genome assembly statistics | | | |
| Total assembled genome size (bp) | 1,767,846,446 | 1,238,768,649 | 1,183,716,478 |
| Total number of scaffolds | 2160 | 45,502 | 78,603 |
| No. of scaffolds ≥ 1000 bp | 2158 | 23,785 | 40,046 |
| No. of scaffolds ≥ 5000 bp | 2107 | 12,169 | 20,461 |
| Longest length (bp) | 45,379,147 | 3,836,756 | 1,105,787 |
| Scaffold N50 (bp) / No. of N50 | 16,975,517/40 | 442,749/706 | 122,458/2581 |
| Contig N50 (bp) / No. of contig N50 | 717,335/434 | 15,754/18,189 | 15,110/18,742 |
| Genomic features | | | |
| GC (%) | 41.21 | 42.73 | 43.29 |
| Predicted protein-coding genes | 20,286 | 18,418 | 19,253 |
| Annotated genes | 18,507 | 15,844 | 16,260 |

*Note*: * means that the genome assembly data of *Eriocheir sinensis* were obtained from NCBI database, <https://www.ncbi.nlm.nih.gov/datasets/genome/GCF_024679095.1/>. NCBI, National Center of Biotechnology Information.
