# Supplementary material for: Genomic Insights into Hybridization and Speciation of Mitten Crabs in the Eriocheir Genus
Source: Genomics Proteomics Bioinformatics. 2025 Sep 15;23(6):qzaf079. doi: 10.1093/gpbjnl/qzaf079 (PMC12996911; doi:10.1093/gpbjnl/qzaf079)
Supplement: qzaf079_Supplementary_Data [file qzaf079_supplementary_data.zip › Table S4.docx]

**Table S4** **Comparison of BUSCO assessment results among *Eriocheir* genomes**

| **Type** | ***E. sinensis****  **(Number/Percent)** | ***E. japonica***  **(Number/Percent)** | ***E. hepuensis***  **(Number/Percent)** |
| --- | --- | --- | --- |
| Complete BUSCOs (C) | 1009/94.65 | 956/89.68 | 934/87.62 |
| Complete and single-copy BUSCOs (S) | 942/88.37 | 911/85.46 | 913/85.65 |
| Complete and duplicated BUSCOs (D) | 67/6.29 | 45/4.22 | 21/1.97 |
| Fragmented BUSCOs (F) | 7/0.66 | 46/4.32 | 64/6.00 |
| Missing BUSCOs (M) | 50/4.69 | 64/6.00 | 68/6.38 |
| Total BUSCO groups searched | 1066/100 | 1066/100 | 1066/100 |

*Note*: * means that the genome assembly data of *Eriocheir sinensis* were obtained from NCBI database, <https://www.ncbi.nlm.nih.gov/datasets/genome/GCF_024679095.1/>. BUSCO, Benchmarking Universal Single-Copy Orthologs.
