# Supplementary material for: Genomic Insights into Hybridization and Speciation of Mitten Crabs in the Eriocheir Genus
Source: Genomics Proteomics Bioinformatics. 2025 Sep 15;23(6):qzaf079. doi: 10.1093/gpbjnl/qzaf079 (PMC12996911; doi:10.1093/gpbjnl/qzaf079)
Supplement: qzaf079_Supplementary_Data [file qzaf079_supplementary_data.zip › Table S5.docx]

**Table S5 Information of sampling localities, and sample size of the 7 populations of *Eriocheir* species**

| **Location** | **Latitude** | **Longitude** | **Site abbreviation** | **Sample identity** | **Sample size** | **Year of collection** |
| --- | --- | --- | --- | --- | --- | --- |
| Vladivostok, Russia | 43.2 | 131.9 | VL | Russian-VL | 32 | 2015 |
| Hokkaido, Japan | 43.1 | 141.6 | HO | Japanese-HO | 45 | 2015 |
| Liao River, China | 41.3 | 122.3 | LR | Chinese-LR | 52 | 2017 |
| Yellow River, China | 38.1 | 119.1 | YeR | Chinese-YeR | 54 | 2017 |
| Yangtze River, China | 31.6 | 121.6 | YaR | Chinese-YaR | 55 | 2017 |
| Min River, China | 26.0 | 119.3 | MR | Min-MR | 22 | 2015 |
| Hepu, China | 21.4 | 109.0 | HP | Hepu-HP | 29 | 2015 |
