# Supplementary material for: Genomic Insights into Hybridization and Speciation of Mitten Crabs in the Eriocheir Genus
Source: Genomics Proteomics Bioinformatics. 2025 Sep 15;23(6):qzaf079. doi: 10.1093/gpbjnl/qzaf079 (PMC12996911; doi:10.1093/gpbjnl/qzaf079)
Supplement: qzaf079_Supplementary_Data [file qzaf079_supplementary_data.zip › Table S7.docx]

**Table S7 SNPs annotation information by SnpEff software**

| **Type** | **Count** | **Percentage** |
| --- | --- | --- |
| Downstream | 6,708,391 | 5.764% |
| Exon | 1,317,392 | 1.132% |
| Missense_variant | 434,715 | 0.373% |
| Synonymous_variant | 887,364 | 0.762% |
| Stop_gained | 8571 | 0.007% |
| Stop_lost | 1076 | 0.001% |
| Start_lost | 1040 | 0.001% |
| Intergenic | 80,318,875 | 69.014% |
| Intron | 21,033,433 | 18.073% |
| Splice_site_acceptor | 1195 | 0.001% |
| Splice_site_donor | 1935 | 0.002% |
| Splice_site_region | 109,853 | 0.094% |
| Upstream | 6,889,109 | 5.919% |
| UTR_5_prime | 41 | 0% |
