# Supplementary material for: Genomic Insights into Hybridization and Speciation of Mitten Crabs in the Eriocheir Genus
Source: Genomics Proteomics Bioinformatics. 2025 Sep 15;23(6):qzaf079. doi: 10.1093/gpbjnl/qzaf079 (PMC12996911; doi:10.1093/gpbjnl/qzaf079)
Supplement: qzaf079_Supplementary_Data [file qzaf079_supplementary_data.zip › Table S8.docx]

**Table S8 Pairwise *Dxy* values identified among different *Eriocheir* populations**

| **Comparison groups** | ***Dxy* value (10^−2^)** |
| --- | --- |
| Chinese-YaR/YeR/LR *vs*. Japanese-HO | 8.02 |
| Chinese-YaR/YeR/LR *vs*. Hepu-HP | 7.55 |
| Chinese-YaR/YeR/LR *vs*. Russian-VL | 7.37 |
| Japanese-HO *vs*. Hepu-HP | 6.81 |
| Japanese-HO *vs*. Russian-VL | 6.93 |
| Hepu-HP *vs*. Russian-VL | 7.17 |
