# Supplementary figures and images for: Genomic Insights into Hybridization and Speciation of Mitten Crabs in the Eriocheir Genus
Source: Genomics Proteomics Bioinformatics. 2025 Sep 15;23(6):qzaf079. doi: 10.1093/gpbjnl/qzaf079 (PMC12996911; doi:10.1093/gpbjnl/qzaf079)

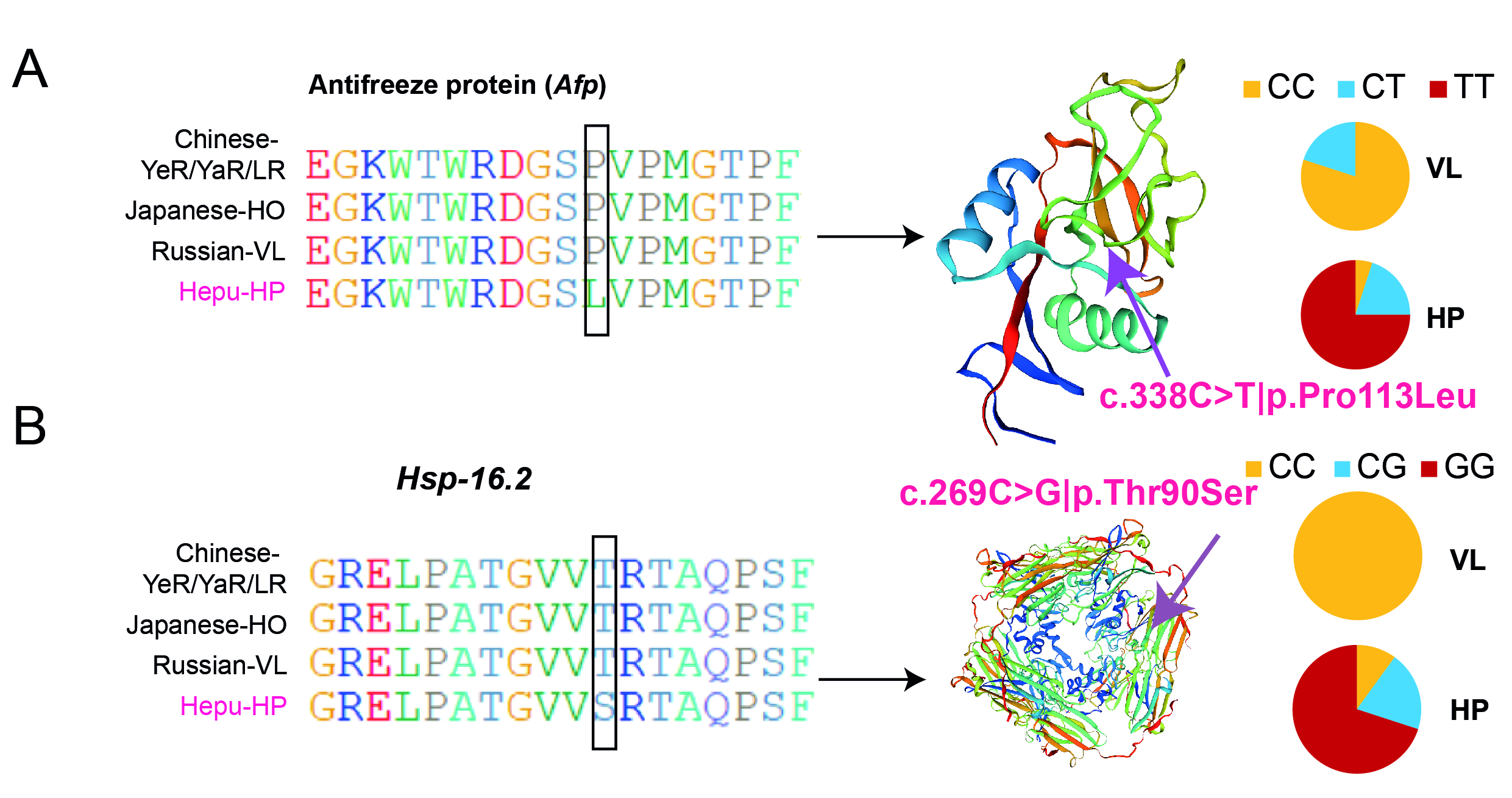

Supplement: qzaf079_Supplementary_Data [file qzaf079_supplementary_data.zip › Figure S10.jpg]

A

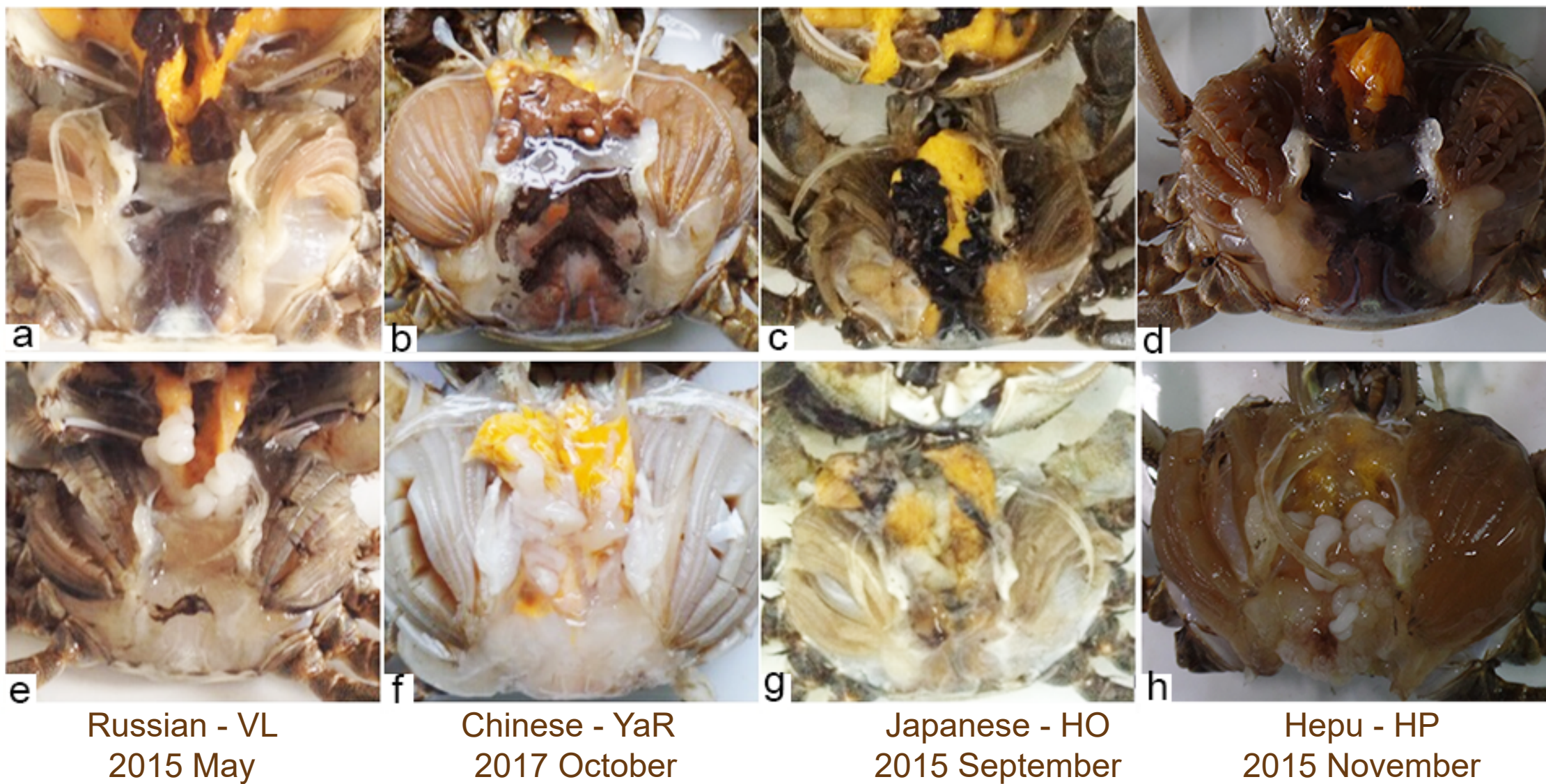

B

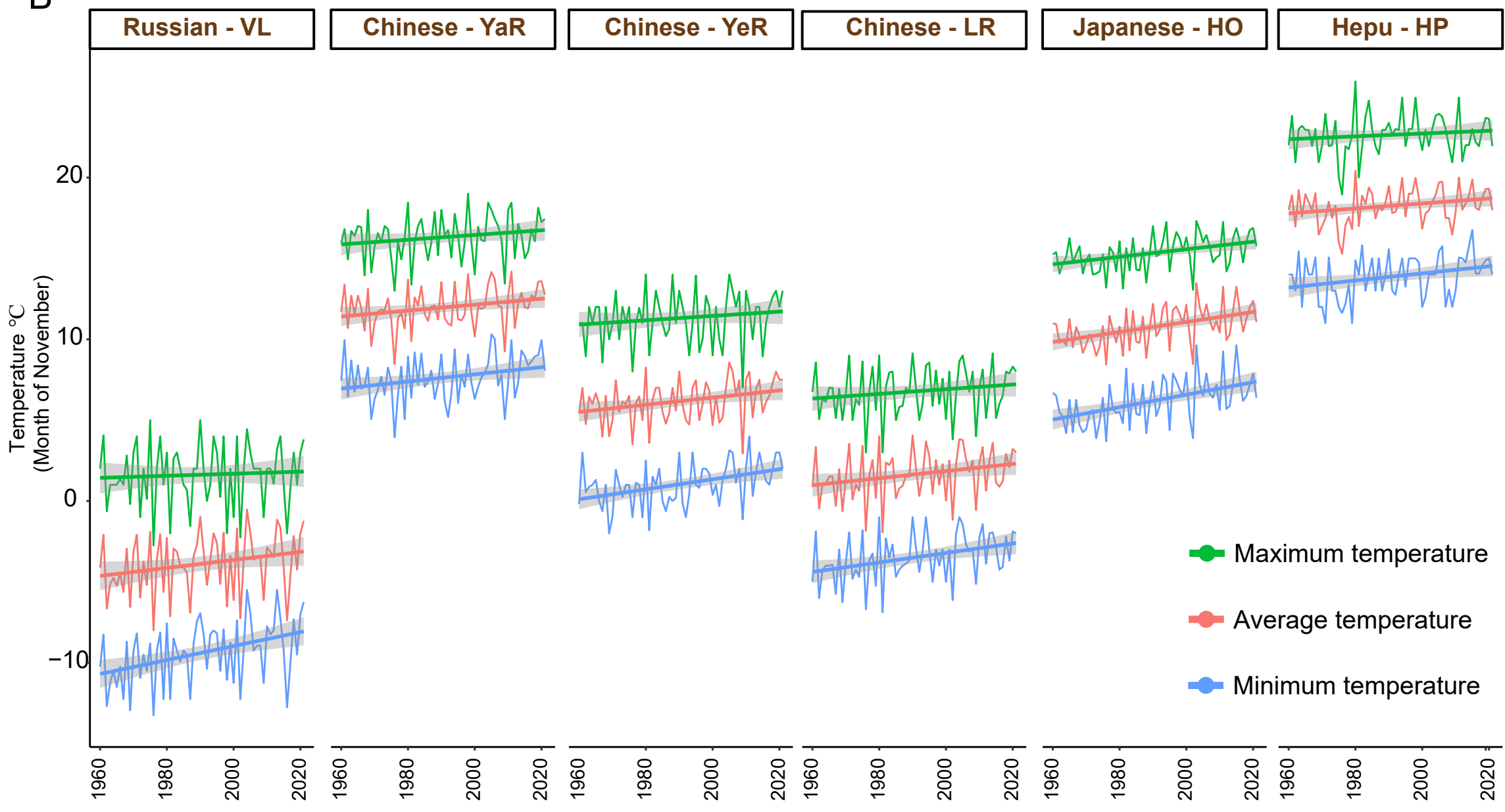

Supplement: qzaf079_Supplementary_Data [file qzaf079_supplementary_data.zip › Figure S2.pdf]

A

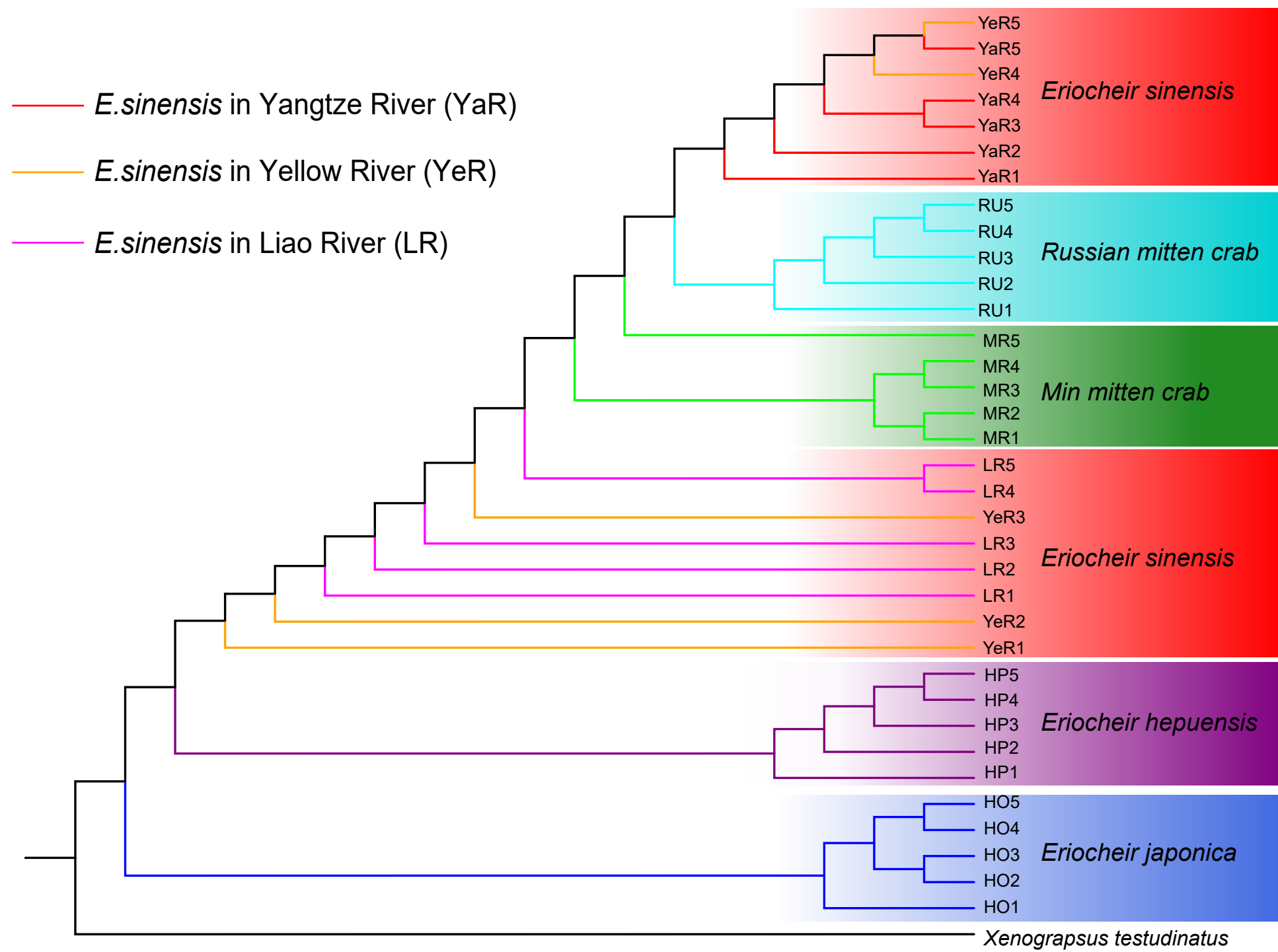

B

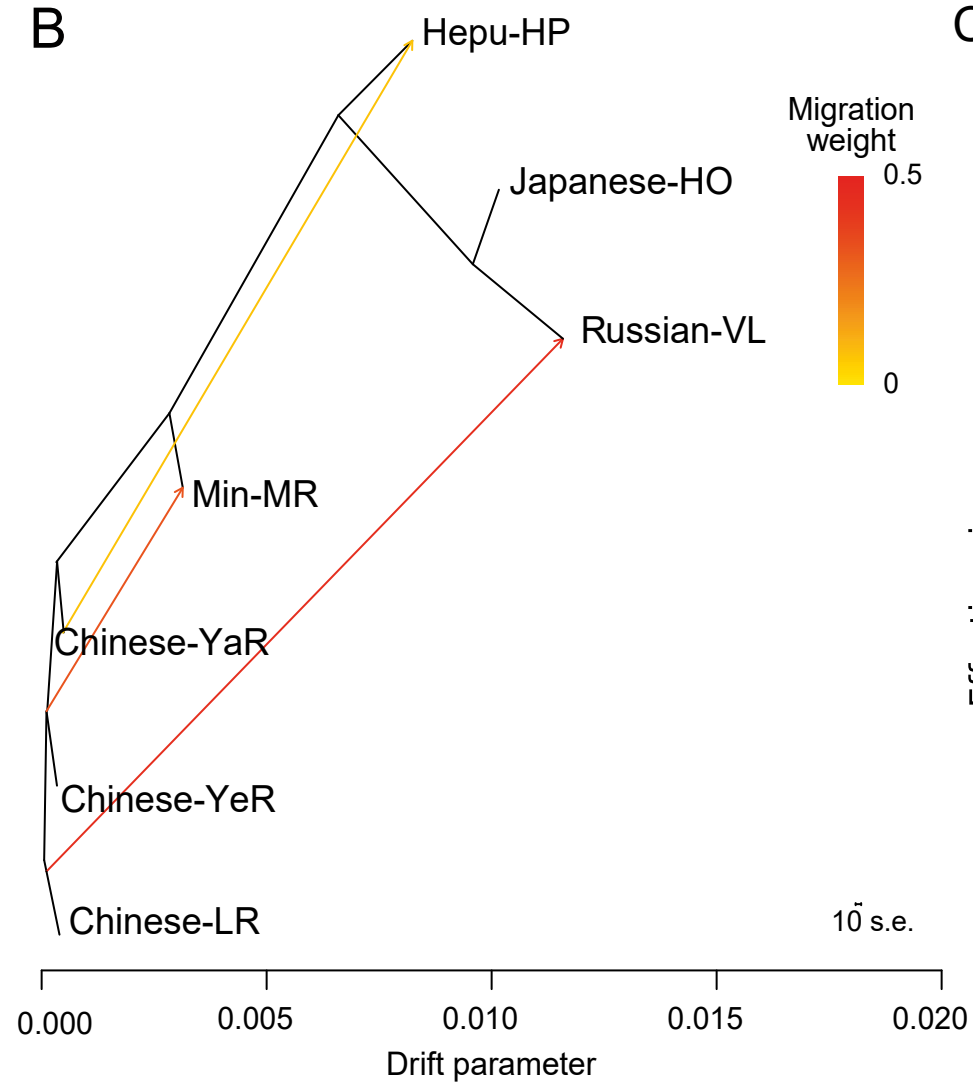

C

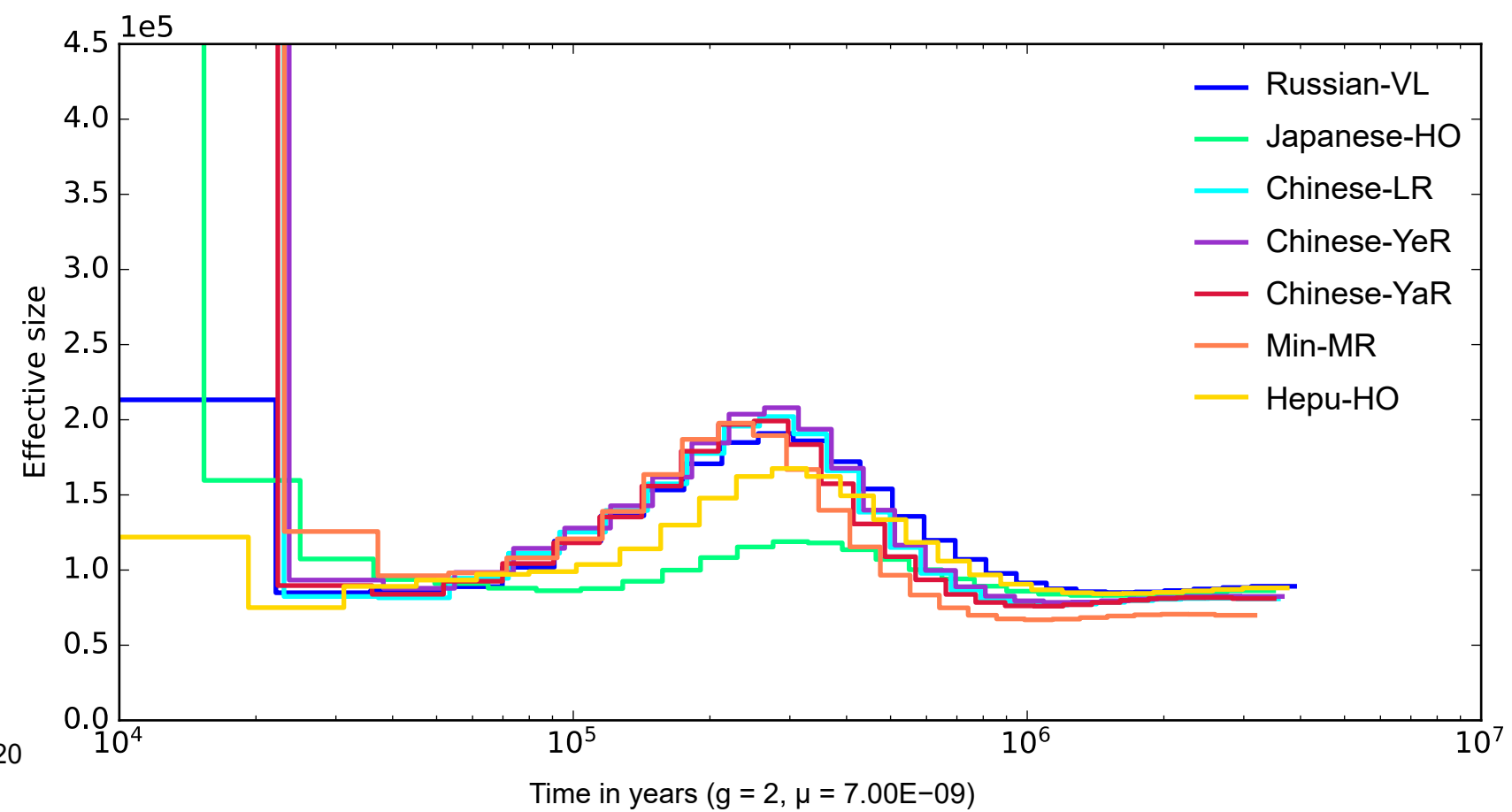

Supplement: qzaf079_Supplementary_Data [file qzaf079_supplementary_data.zip › Figure S3.pdf]

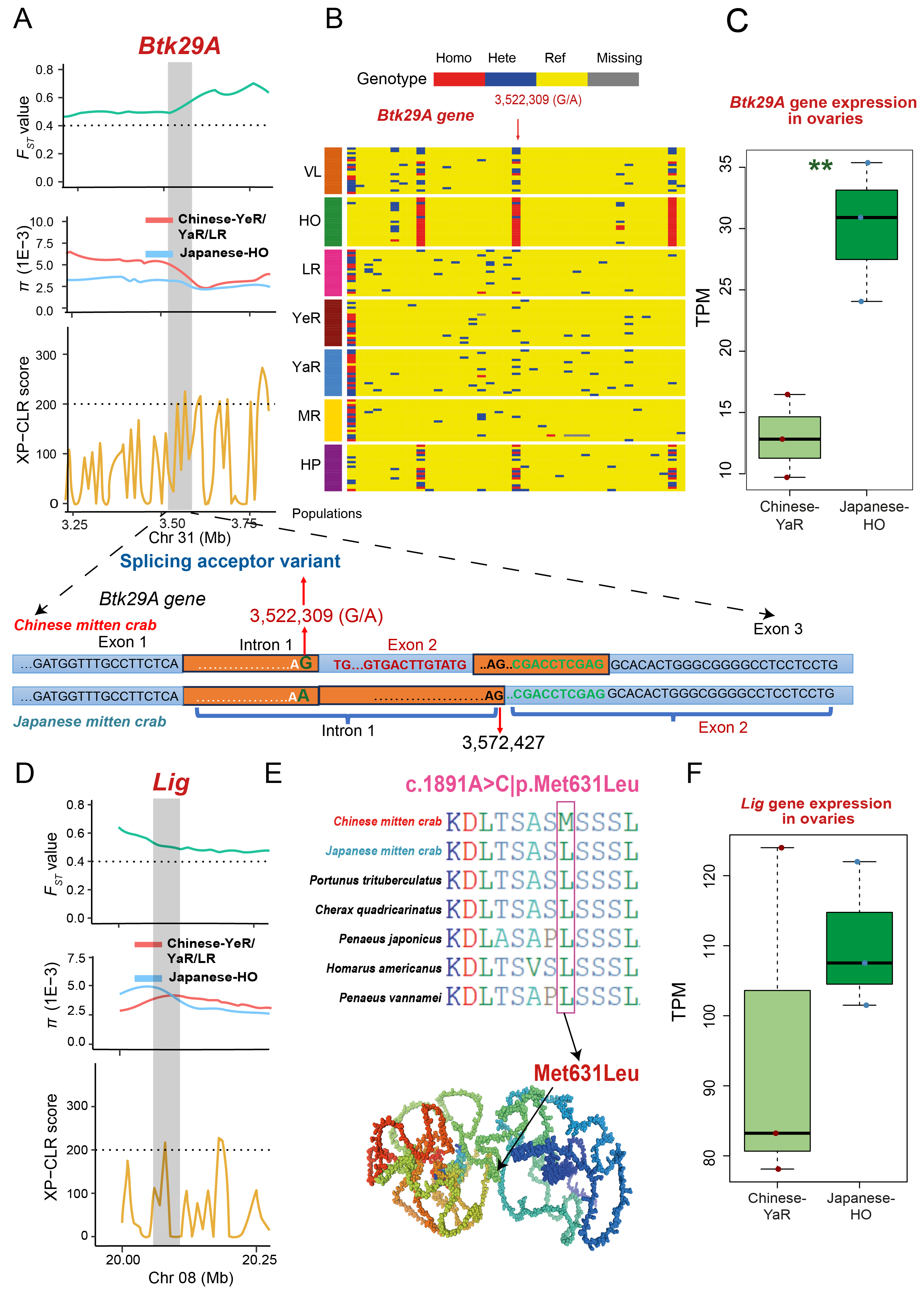

Supplement: qzaf079_Supplementary_Data [file qzaf079_supplementary_data.zip › Figure S5.jpg]

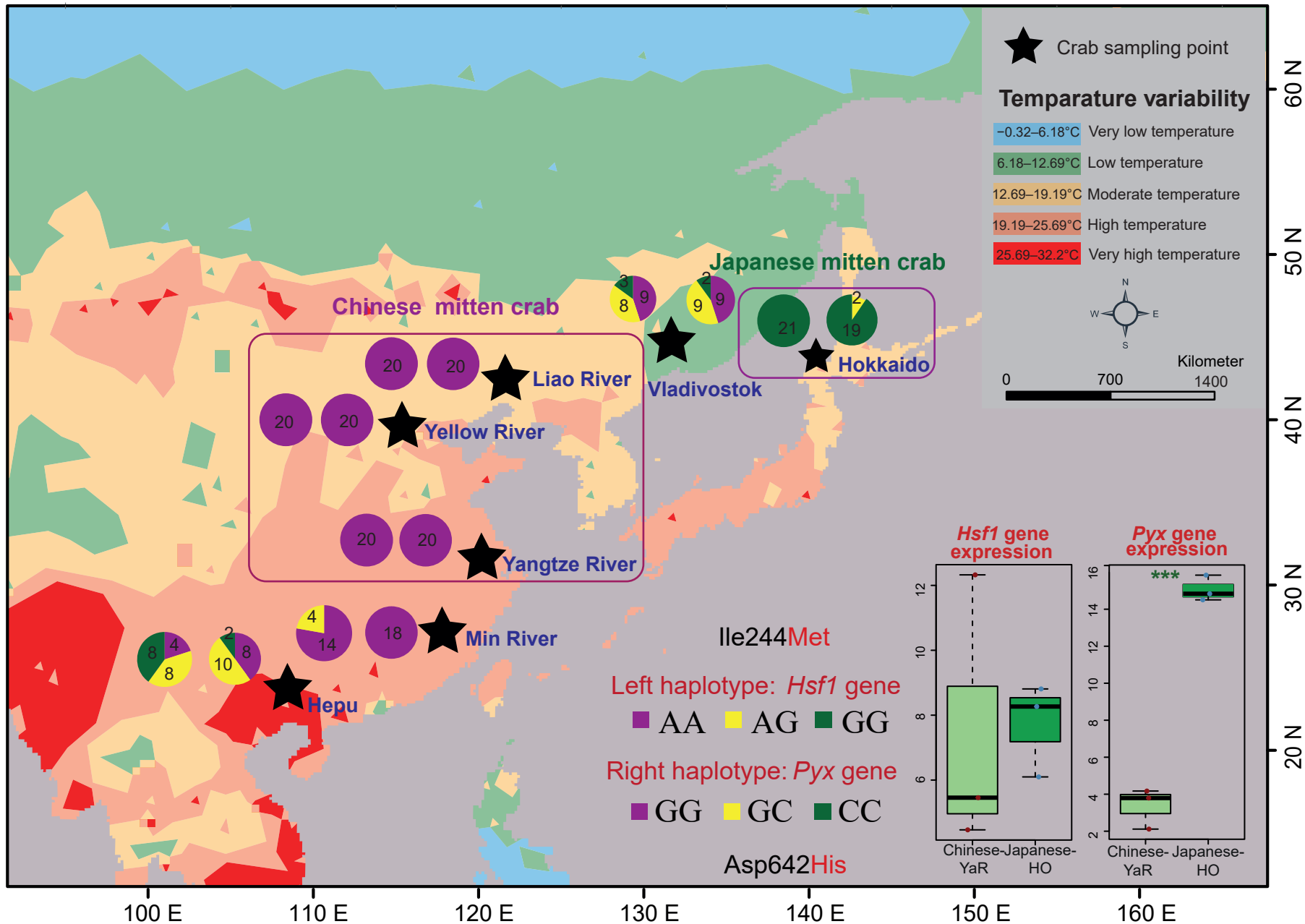

Supplement: qzaf079_Supplementary_Data [file qzaf079_supplementary_data.zip › Figure S6.pdf]

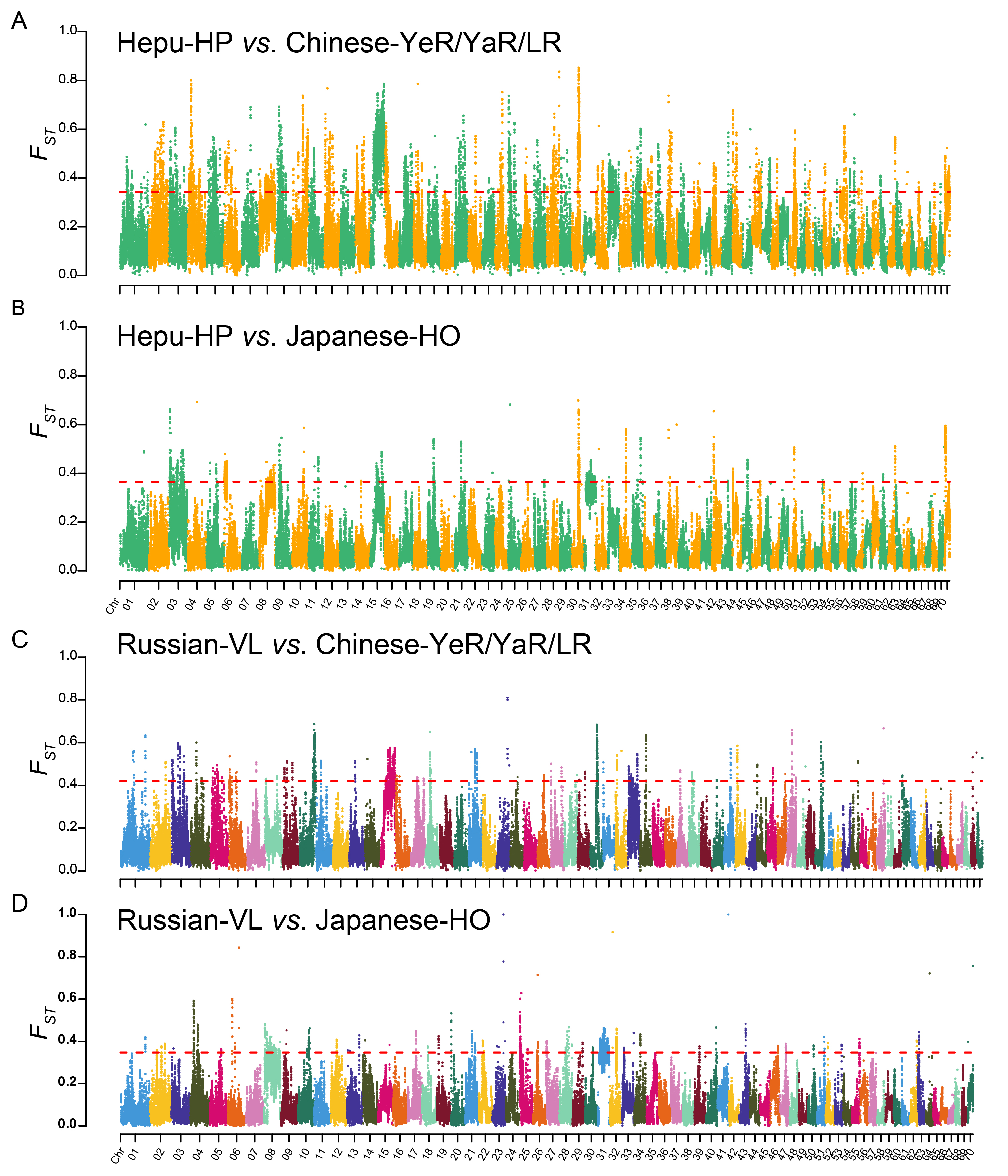

Supplement: qzaf079_Supplementary_Data [file qzaf079_supplementary_data.zip › Figure S7.jpg]

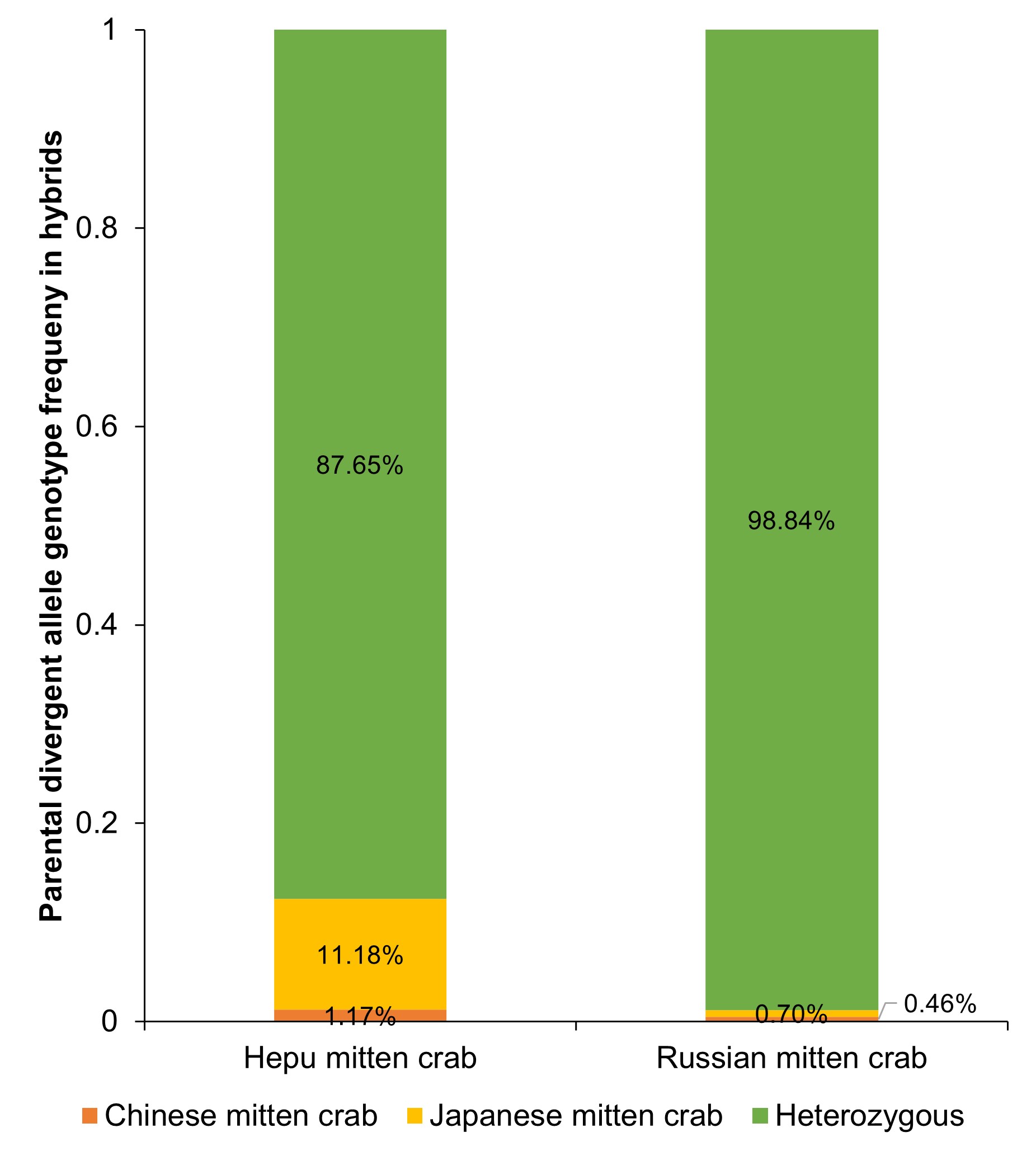

Supplement: qzaf079_Supplementary_Data [file qzaf079_supplementary_data.zip › Figure S8.jpg]

Homo Hete Ref Missing

Genotype

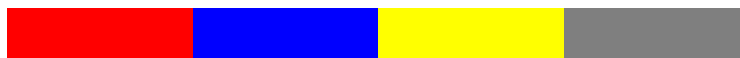

*BIRC6* exon 7

*BAP31* exon1

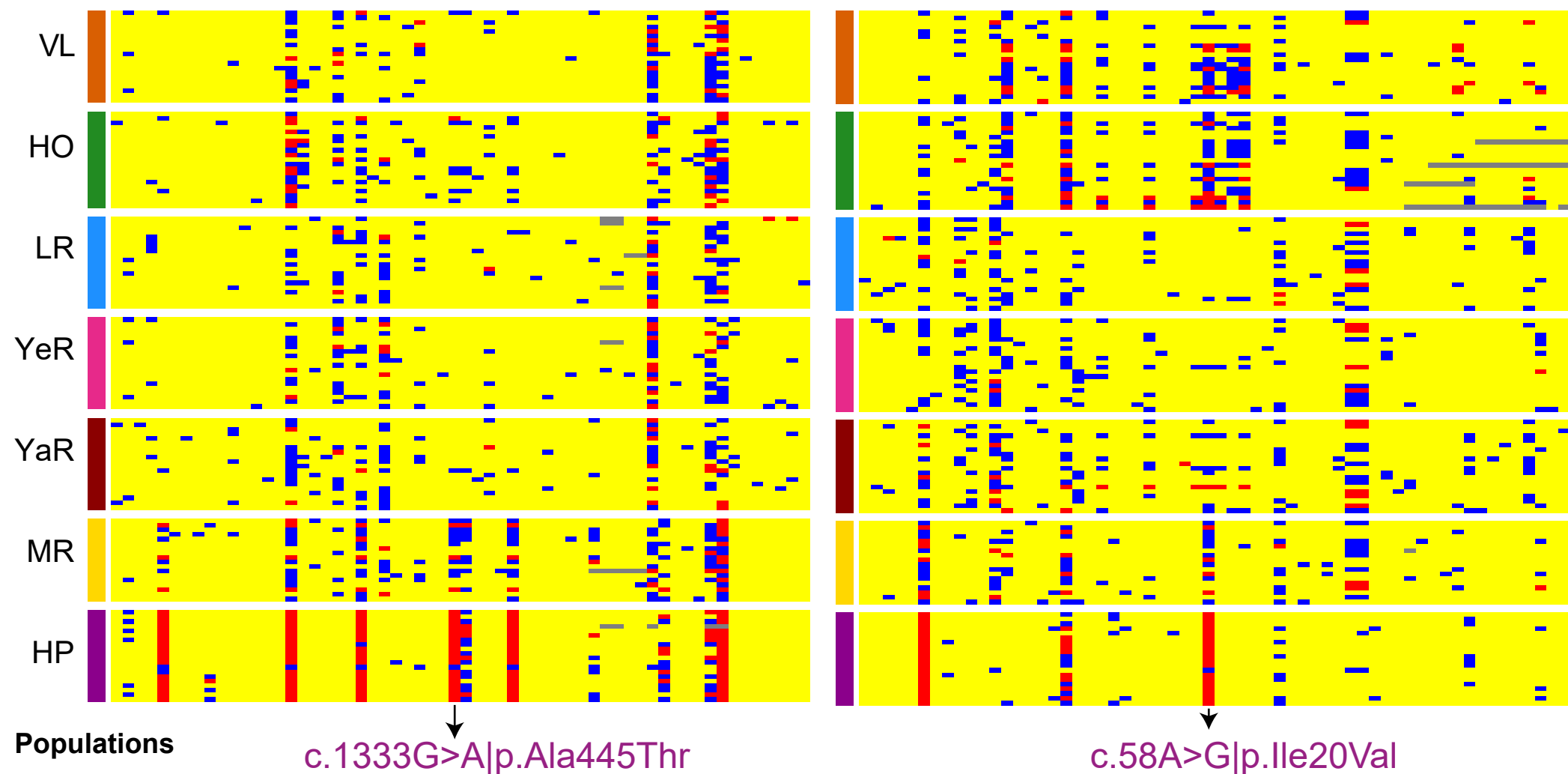

*Poxn* exon 6

*Shc1* exon 8

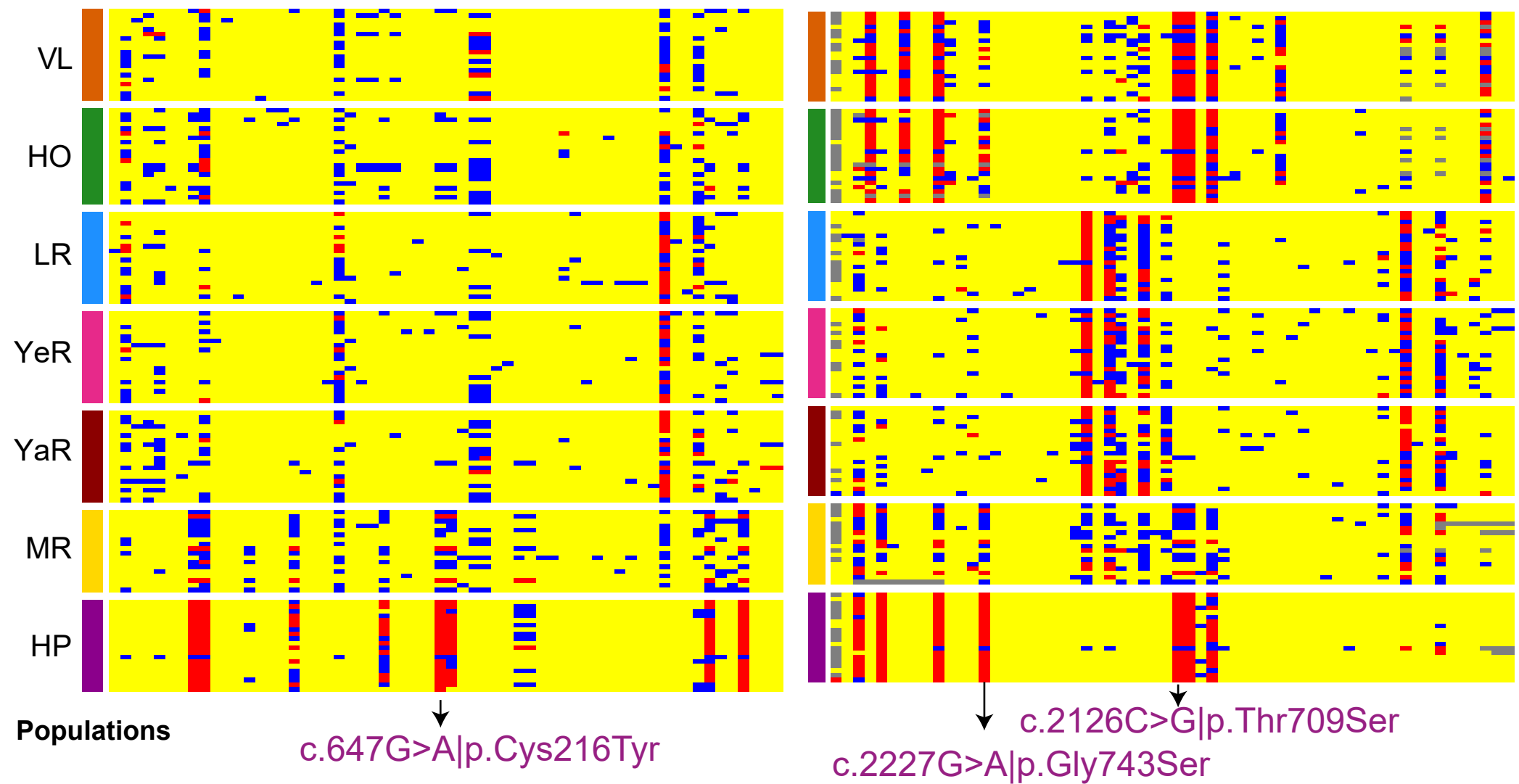

Supplement: qzaf079_Supplementary_Data [file qzaf079_supplementary_data.zip › Figure S9.pdf]
